# Sexual Selection Associated With an Aggressive Male Phenotype Reduces Population Size and Hinders Population Recovery After Heat Stress

**DOI:** 10.1111/ele.70377

**Published:** 2026-04-17

**Authors:** Neha Pandey, Neelam Porwal, Jonathan M. Parrett, Jacek Radwan, Robert J. Knell, Tom C. Cameron

**Affiliations:** ^1^ Evolutionary Biology Group, Faculty of Biology Adam Mickiewicz University Poznań Poland; ^2^ Faculty of Science and Engineering, School of Natural Sciences University of Hull Hull UK; ^3^ School of Life Sciences University of Essex Colchester UK

**Keywords:** environmental perturbation, heat stress, population dynamics, population recovery, population stability, resilience, *Sancassania berlesei*, sexual selection

## Abstract

Population recovery following environmental stress is known to depend on demographic structure, life‐history and evolutionary dynamics. However, it is unclear how traits shaped by sexual selection affect population dynamics and recovery. We examined this by manipulating presence/absence of males expressing either a non‐aggressive ‘scrambler’ phenotype or an aggressive and lethally armed ‘fighter’ phenotype in soil mite populations of different size. We experimentally altered the male phenotype in populations, subjected them to heat stress, and analysed their population dynamics and recovery. We show that populations with fighter males exhibited (i) reduced population size and stability, (ii) greater decline in response to heat stress in larger populations, (iii) higher rate of growth and (iv) incomplete population recovery. Such reduced population stability and recovery linked with armed and aggressive phenotypes underlines the importance of sexual selection in mediating population dynamics and resilience to environmental change with implications for managing natural populations.

## Graphical abstract

Using experiments on soil mites, we show that sexual selection associated with an armed and aggressive male phenotype can reduce population size and stability, which lowers their resilience against acute heat stress. These effects linked with armed and aggressive phenotypes underline the importance of sexual selection in mediating population dynamics and resilience to environmental change.

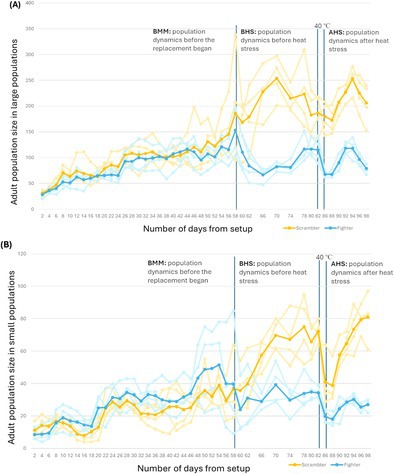

## 1 Introduction

The ability of sexual selection to drive evolutionary changes through mate choice or competition for mating has always evoked considerable interest (Darwin 1871; Fisher 1930). A large body of literature has accumulated on how sexual selection facilitates the evolution of many traits (Tuschhoff and Wiens 2023; Emlen 2008), and on its fitness costs and benefits (Candolin and Heuschele 2008; Rowe and Rundle 2021). However, the role of sexual selection in driving the dynamics of population size and growth across generations remains relatively unclear. As sexually reproducing populations experience some form of sexual selection, understanding the demographic consequences of this selective force and its interactions with natural selection has important implications for predicting population dynamics and persistence in the light of contemporary environmental changes.

There are several ecological consequences of sexual selection which could influence population dynamics. Under strong intra‐sexual competition, males face increased mortality due to direct injury from contests or resource costs of extravagant display (Giery and Layman 2019). Further, male competition can affect female fitness through effects on fecundity or mortality risk arising from harassment or inter‐sexual aggression (Le Boeuf and Mesnick 1990, Arnqvist and Rowe 2002, Le Galliard et al. 2005, 2008, but see Fromonteil et al. 2023). Such proximate effects on mortality and fecundity can change adult sex ratios and population density, impacting population growth (Rankin and Kokko 2007; Rankin et al. 2011; Eberhart‐Phillips et al. 2017; Brides et al. 2017). Moreover, condition‐dependent expression of sexually selected traits (e.g., weaponry) and environment‐induced changes in sex‐ratio can also exacerbate inter‐sexual conflict and mate harm, increasing extinction risk (Kokko and Brooks 2003; Rankin et al. 2011; Flintham et al. 2023). Such feedback between sexual selection and environment can affect population growth through immediate differential effects on demographic classes (Le Boeuf and Mesnick 1990) which may impact population resilience (i.e., resistance and recovery) (*sensu* Capdevila et al. 2020), after environmental stress.

In addition to these short‐term impacts, sexual selection could shape population dynamics through evolutionary changes across generations. The reduction in effective population size caused by conflict‐linked mortality or skewed reproductive success can increase inbreeding depression and drift‐mediated loss of genetic variability, thus increasing extinction risk (Clutton‐Brock et al. 1997; Kokko and Brooks 2003; Rankin and Kokko 2007). Contrastingly, sexual selection could enhance population persistence by purging mutation load (Lorch et al. 2003; Whitlock and Agrawal 2009; Lumley et al. 2015; Dugand et al. 2019; Parrett et al. 2022) and promoting beneficial alleles (Whitlock 2000; Parrett and Knell 2018).

While there is limited empirical understanding of the effects of sexual selection on different aspects of population dynamics, studies on extinctions indicate inconsistent effects. Empirical studies show substantial differences in the impacts on extinction risk and adaptation to environmental change, with both positive (e.g., increased persistence and adaptive advantage: Plesnar‐Bielak et al. 2012, Lumley et al. 2015, Jacomb et al. 2016, Porwal et al. 2025) and negative outcomes (e.g., more extinctions and maladaptive responses: Martins et al. 2018, Łukasiewicz et al. 2023). Importantly, these outcomes are likely to critically depend on population size. For example, in small populations, higher male mortality in sexually selected populations can increase demographic stochasticity and increase extinction risk, potentially leading to further loss of genetic diversity, with possible effects on long‐term persistence. In contrasts, larger populations may have an advantage due to faster adaptation rates (Martínez‐Ruiz and Knell 2017). Despite such non‐trivial impacts of sexual selection, its effects on population dynamics, stability and growth rates have rarely been examined.

Contemporary natural populations are increasingly experiencing environmental change, with long‐term shifts in thermal environments being commonly reported, alongside the increasing frequency of acute thermal stress in the form of heatwaves (Fuller et al. 2016; Spooner et al. 2018; Cunningham et al. 2021). To our knowledge, there has been no empirical examination of the role of sexual selection in mediating the dynamics and recovery of populations of different sizes, especially in response to acute environmental change such as heat stress. When populations experience environmental stress, their population structure is perturbed and their dynamics can destabilize (Cameron and Benton 2004; Ozgul et al. 2012; Capdevila et al. 2020). While several studies have examined population responses to stress (Lindström and Kokko 2002; Beckerman et al. 2003; Benton and Beckerman 2005; Ozgul et al. 2012), it remains unclear whether such responses are mediated by sexual selection. As the strength and mode of sexual selection likely vary in natural populations, an experimental understanding of the role of sexual selection could improve the predictive knowledge of population dynamics experiencing environmental change (see Berger and Liljestrand‐Rönn 2024 for effects of monogamy/polygamy).

Here, we investigate the consequences of the presence or absence of a sexually selected phenotype expressing lethal weaponry and aggression on population dynamics. Such phenotypes are a commonly observed outcome of sexual selection (Darwin 1871; Andersson 1994) and have consequences for survival of their bearers (Marler and Moore 1988; Bro‐Jørgensen 2014) as well as other males and females (Le Boeuf and Mesnick 1990; Arnqvist and Rowe 2002; Le Galliard et al. 2005, 2008). For our investigation, we leverage a soil mite model system, *Sancassania berlesei*, that has dimorphic males: one morph—the fighters—have exaggerated sexually selected male weapons in the form of a thickened third pair of legs, which are absent in the other morph, scramblers (Radwan 1993). To secure mating access through contests with rival males, fighter males aggressively use this weaponry to kill competing males (Radwan 1993) or cause major harm to males as well as nearby females (Łukasik 2010). Thus, it is expected that the presence of fighter males may have short‐ and long‐term demographic consequences via increased mortality. In contrast, the scrambler males compete without the fighting armaments (Radwan 1993). Crucially, fighter expression is condition‐dependent, with heavier juvenile males emerging as fighters (Radwan et al. 2002). In small populations, fighter males can monopolize the females by killing all rivals (Radwan 1993), but in large populations this is not possible, making the effects of population size important. In addition to mortality arising from strong male–male competition, both male morphs may also reduce female fecundity and longevity when they attempt multiple matings (Radwan and Rysińska 1999; Łukasiewicz 2020), while the fighters can additionally increase the mortality of females by cannibalizing them under food scarcity (Łukasik 2010). Considering these potential demographic impacts of this sexually selected phenotype, we examined if the dynamics of populations containing this lethally armed phenotype differ from those without it, especially in their response to environmental stress.

Specifically, we experimentally manipulated the presence of the fighter‐male morph, heat stress and population size, to examine if pre‐copulatory intra‐sexual selection associated with this phenotype (i) affects population size, growth rate and dynamics, (ii) impacts inter‐sexual conflict, (iii) affects the demographic response to an environmental perturbation and shapes the resilience of these populations after experiencing the perturbation. Further, we examined (iv) whether any observed effects differ between small and large populations; where in small populations, fighters can maximize access to females by killing rivals, which may be difficult in large populations (Radwan 1993). We hypothesized that pre‐copulatory sexual selection, arising from the presence of aggressive males will reduce population size, with stronger reductions in male than female population size. We predict that such sexual selection may reduce maximum (*r*_*max*_) and realized population growth rates (*r*) by limiting female fertility due to the decline in male numbers (Vasilieva and Tchabovsky 2015) and possibly also via direct detrimental effect of male aggression on females (Le Boeuf and Mesnick 1990; Łukasik 2010). Crucially, we expect lower population stability (greater fluctuations over time within a population) under higher male aggression, because the aggressive phenotype increases the likelihood of stronger stochastic interactions with food availability– for instance, pulses of fighter male recruitment can reduce male population size and promote over‐compensatory recruitment dynamics (Ozgul et al. 2012; Beckerman et al. 2003). Finally, we predict a slower or incomplete recovery after heat stress in populations with the aggressive male phenotype, because adult mortality was expected to increase due to the presence of this male morph. We expected this effect of the fighter male‐morph presence on recovery to be more evident in small populations due to higher demographic stochasticity.

## 2 Methodology

### 2.1 Population Dynamics Experiment

**FIGURE 1:**
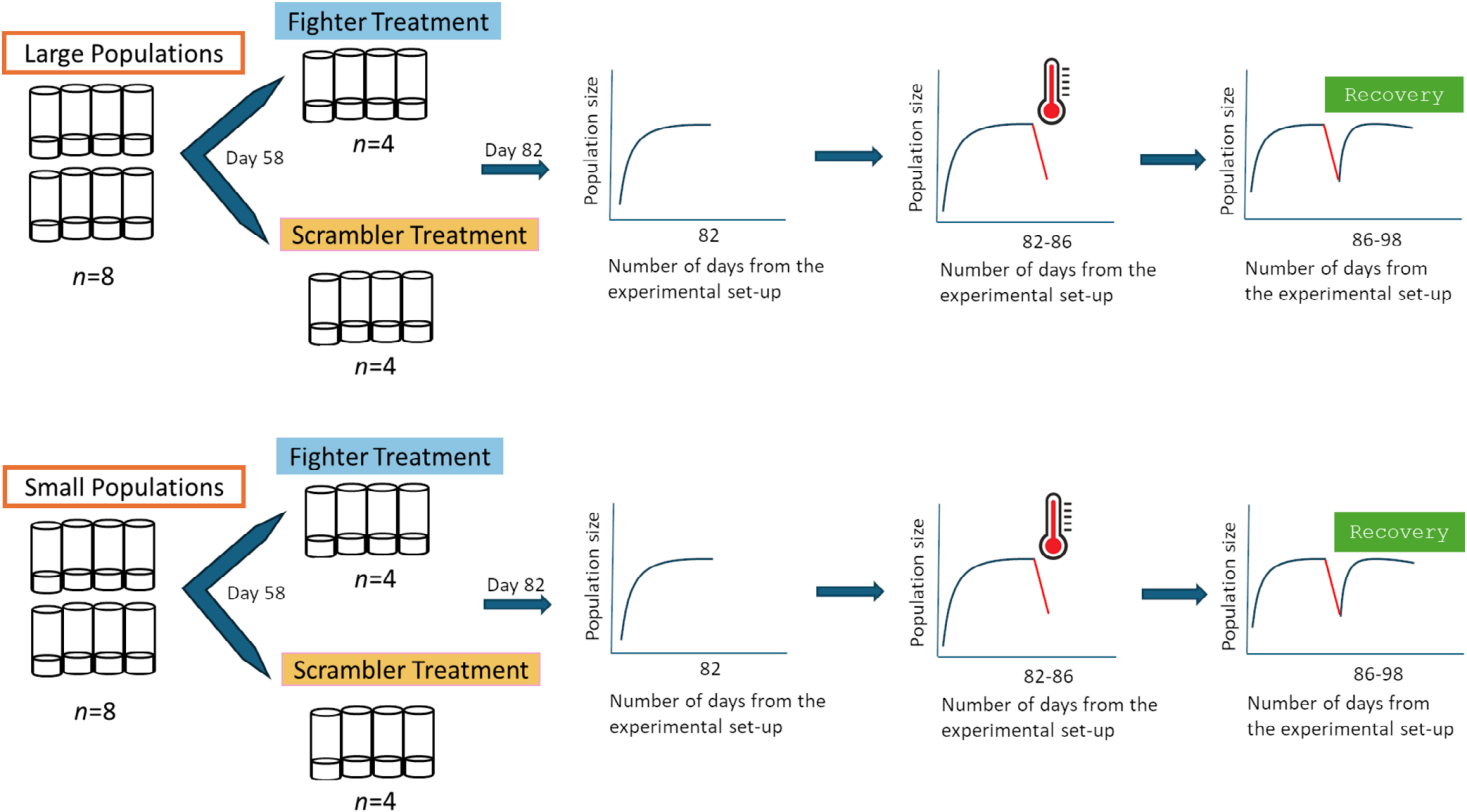
Detailed schematic of the protocol of the population dynamics experiment. From Day 58 onwards the fighter and scrambler treatments were established by replacing the males with the right types of morphs from the stock population. On Day 82 from the setup, all the populations (including stocks used for replacement) were given heat stress at 40°C for 90 h, after which the survivorship and recovery were recorded.

The experimental protocol is summarized in Figure 1. We derived 16 replicate populations from the stock population (see Supporting Information [SI] for stock origin and maintenance) and allocated eight each to two levels of population sizes maintained during the experiment: small and large population sizes. The large populations were established with a spoonful (spoon dimensions: 9.6 × 4.8 mm) of mites (all life‐stages), while small size populations were established with one quarter spoonful of mites. We started with these quantities and all the life‐stages, as they are expected to stabilize faster with controlled food supply (may take up to 40 days: Cameron and Benton 2004). Based on a pilot, large populations were maintained by supplying a greater biomass of food (40 standard‐sized yeast balls) than for small populations (10 standard‐sized yeast balls), which should support roughly 150 adults in large populations and 40 adults in small populations (SI). While physical space (diameter of vial 2.4 cm^2^) available to mites was the same in both population size treatments, mites clustered on patches of food provided, so effective density/food patch occupied was likely similar for populations of different size. All the replicate populations were given food every second day.

With the intention to establish sexual selection treatments with or without the effects of aggressive male morphs, we kept only the aggressive fighter males in the fighter treatment and scrambler males in the scrambler treatment. These two treatments are expected to differ in the pre‐copulatory sexual selection arising due to the aggressive male morph. The male‐morph treatments were established from Day 58 onwards (as the populations were expected to be stabilized) by replacing newly emerged scrambler males with newly emerged fighters from the stock population for the fighter treatment (Figure 1). The fighters that were introduced from the stocks were better fed and thus visibly bigger than the males already present (due to limited food availability in the population vials). This was necessary because food‐limited juveniles rarely emerge into fighters (Radwan 1995, Figure S1). Since the introduction of new fighter males from the stock population could potentially introduce noise to the population dynamics and affect size, structure, or genetic composition of the populations (including reduction in inbreeding), we treated the scrambler treatment populations in a similar way by replacing the same percentage (see SI) of newly emerged males from the scrambler vials with newly emerged scrambler males from stock. The replacements were done on every day of the week, except on weekends. While we cannot account for the loss of genetic variance in experimental populations in our study as we replaced the male morphs from stock, our experiment on populations of distinct sizes can inform about the effects of demographic stochasticity.

On Day 82 since the start of the experiment, all the replicates (including stock used for replacements) were given a heat stress, with an acute increase in temperature to 40°C for 90 h (temperature and duration were decided using a pilot experiment). This day for the heat stress perturbation was chosen to let the population stabilize after the male morph manipulations while leaving enough time to study the response to the heat treatment. The egg‐to‐adult development time in this species is 10–11 days at 23°C. A census of tritonymphs (last juvenile stage), males (both fighter and scrambler), and females was undertaken in all populations every alternate day, except for the period between Day 62 and Day 78, during which census was done every 4 days. All replicate populations were maintained at 23°C, except during the heat stress period. Finally, we conducted a fecundity assay on the females collected from these populations after the population dynamics experiment (see SI).

### 2.2 Statistical Analyses

**FIGURE 2:**
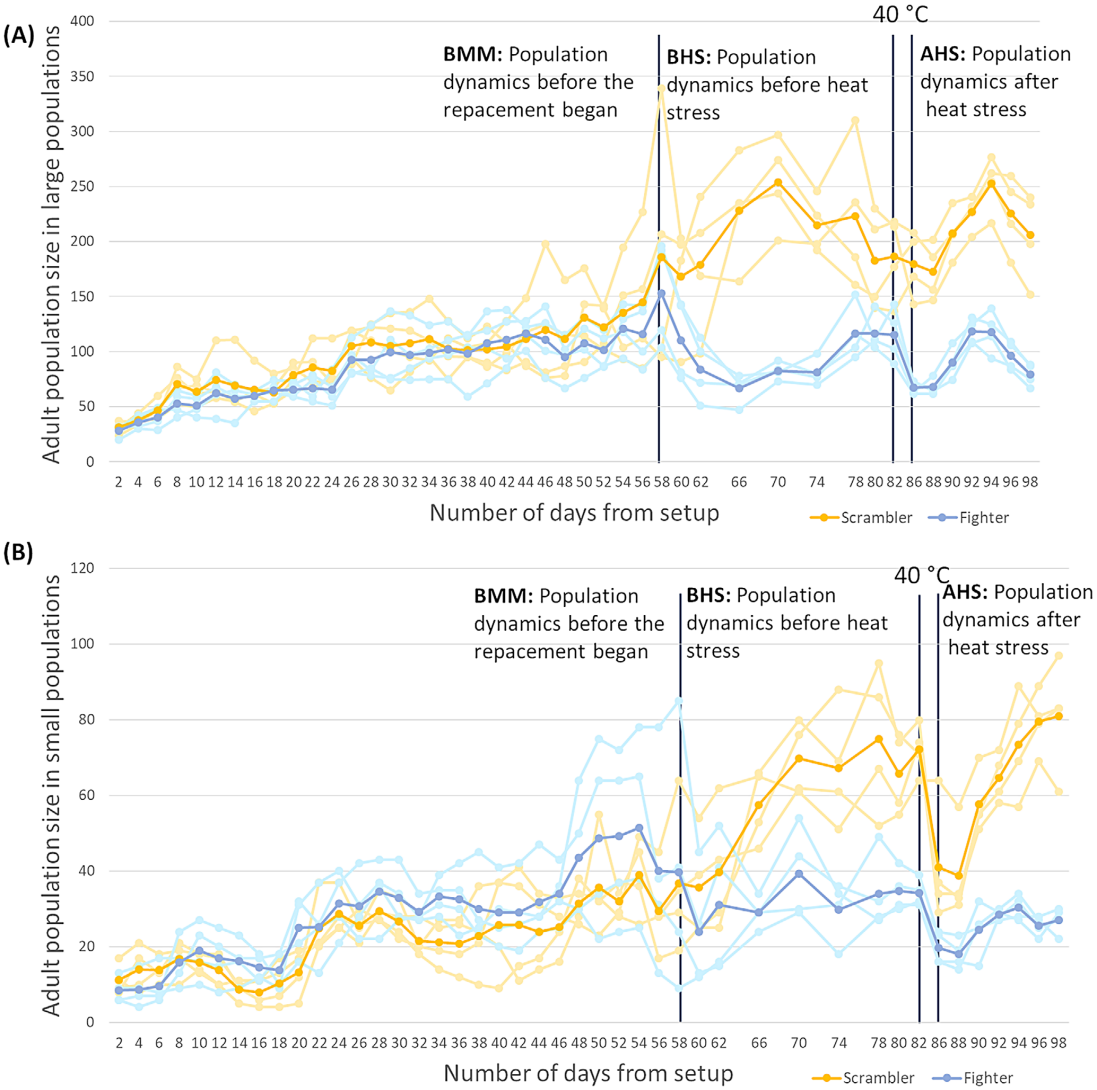
Adult population size time series in (A) large and (B) small populations of scrambler and fighter treatments. The dark blue colour is the mean adult population size in the fighter treatment, and the dark orange colour is the mean adult population size in the scrambler treatment, while all the lighter colours show adult population size in the respective replicate populations. The fighter and scrambler treatments were established by replacing male morphs from the stock populations from Day 58 from the experiment setup. On Day 82 from setup, the populations were given a heat stress for 90 h at 40°C. Population census were done on every alternate day except for the period between Day 62 and Day 78 when census was done every 4 days.

The time‐series from our study populations are shown in Figure 2. We divided our time‐series data in three time periods, that is, before morph manipulation (BMM, Day 2–58), before heat stress but after manipulations began (BHS, Day 58–82), and after heat stress (AHS, Day 86–98). We used linear mixed and generalized linear mixed‐effects models (GLMM, when errors were overdispersed), using the ‘glmmTMB’ package (Brooks et al. 2017), to examine the effects of male‐morph treatment (fighter/scrambler), population size (large/small), time period, and the interactions between them on the following response variables: adult population size, tritonymph population size, male/female survivorship after male morph replacement, sex ratio, decline in adult population size, CV_adult‐population‐size_, CV_total‐population‐size_, mean‐corrected variability_adult‐population‐size_, mean‐corrected variability_total‐population‐size_ (see below), *r*_*max*_, *r*, percentage recovery after heat stress and fecundity (results tables in SI). The replicate population identity was the random effect. We visually examined diagnostic plots to check how well model assumptions were met, using the DHARMa package (Brooks et al. 2024, Hartig 2024).

We analysed census data on adults, tritonymphs (reflecting recruitment rate), and sex ratio from the time the male manipulations were introduced (Day 58th), using generalized additive mixed models (GAMMs) fitted using the ‘*mgcv*’ package (Wood and Wood 2015), with a smoothing term for the time component (days) and population as the random factor. These models were originally fitted using ML estimation to allow comparison using likelihood ratio tests, and then the final selected model was re‐fitted using REML for inference (Zuur et al. 2009). For the response variables tritonymph population size and adult sex ratio (male to female ratio), we used a Gaussian error structure to compare the fighter and scrambler treatments separately for the BHS and AHS periods. For adult population size, we used Poisson (BHS) and quasi‐Poisson (AHS) error structures.

Next, we quantified population stability by calculating the coefficient of variation (CV) in population size across time.

However, since CV can be biased when standard deviation does not linearly increase with mean (Supplement text b), we additionally calculated mean‐corrected variability (Hosken et al. 2025) using the deviation from this linear expectation as follows:

$$\text{Mean}-\text{corrected variability}=\mathrm{Log}\;\left(\frac{\text{observed standard deviation in population size}}{\text{expected standard deviation in population size}}\right)$$

We used GLMM with a gamma error structure to analyse CV, a gaussian error structure to analyse mean‐corrected variability, and a binomial error structure to analyse fecundity. To test if population stability was affected by the prevalence of aggressive male phenotype, we compared mean‐corrected variability in adult and total population size (adult population size + tritonymph population size) from Day 34 to Day 58 (BMM) and Day 58 to Day 82 (BHS). We also compared mean‐corrected variability in the BHS and AHS periods to examine how heat stress affected population stability. For both the BHS and AHS periods, at least one generation (12 days) of data was analysed. The *r*_*max*_ (max *N*_*t+1*_/*N*_*t*_) and *r* (*N*_*t+4*_/*N*_*t*_), in fighter treatment versus scrambler treatment was compared in BHS and AHS time period. We calculated *r* with *N*_*t+4*_/*N*_*t*_ to cover at least one generation during this period. Thus, for these attributes, we had an interaction of treatment and population size with the time period (BHS/AHS).

To assess the impact of introducing fighter males or removing them on survivorship of males and females, we compared male and female count on Day 58 and Day 60. We examined the effect of heat stress on survivorship by analysing the decline in adults after heat stress. Finally, we quantified percentage recovery after the heat stress, as the final population size on the last day (Day 98) as a percentage of population size prior to heat stress.

We ran the models with all fixed effects and their interactions, but in the results, we report only the minimum adequate models retaining significant fixed effects. To obtain the minimum adequate model, we started with the full model and used stepwise simplification with likelihood ratio tests to remove non‐significant interactions for the LMs and GLMMs. All statistics were run in R version 4.5.1 (R Core Team 2025).

## 3 Results

### 3.1 Presence of Aggressive Male Morph Affects Population Size and Sex Ratio

**FIGURE 3:**
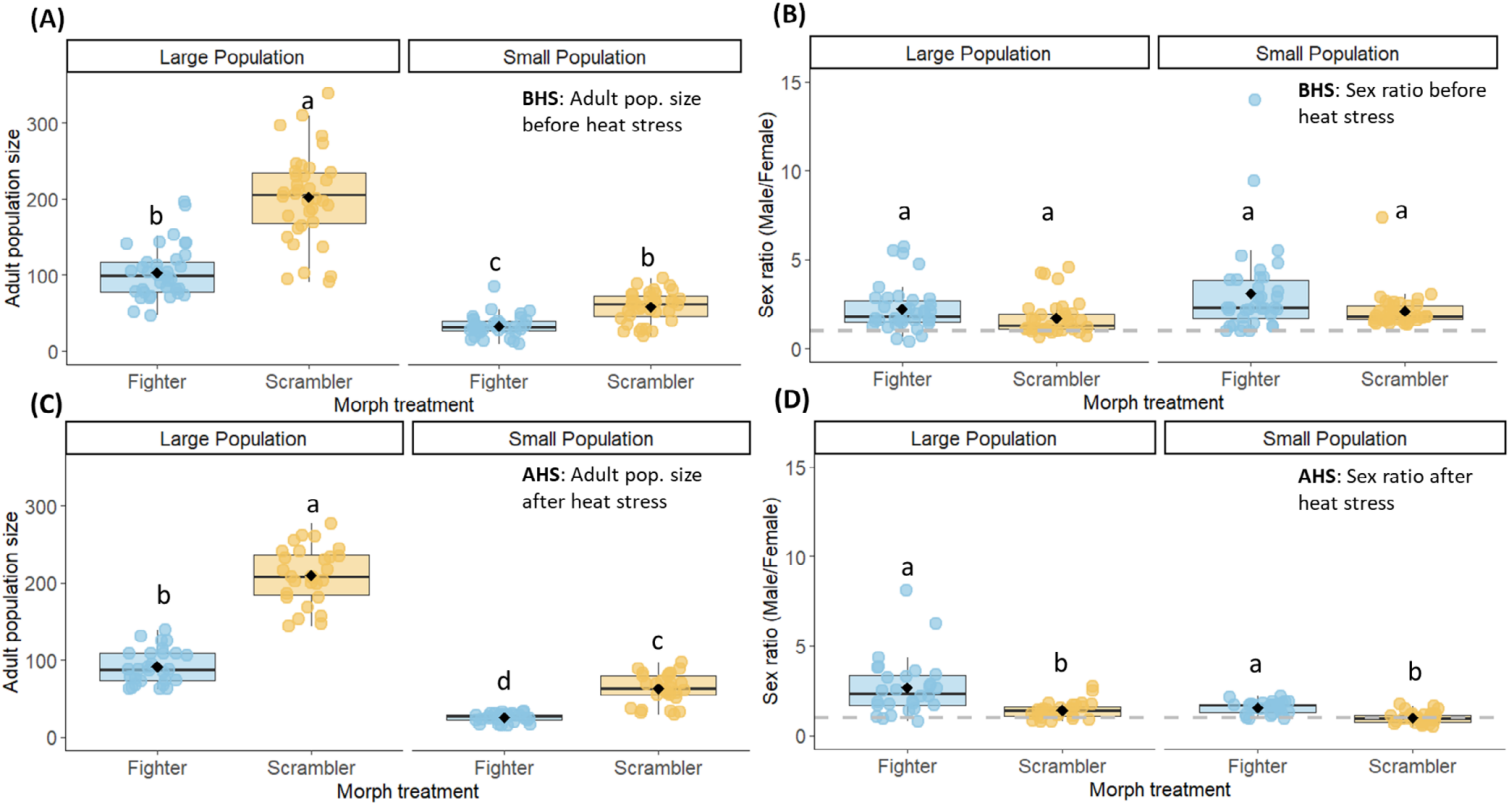
Adult population size (A) and sex ratio variation (B) in the before heat stress (BHS) time period, and adult population size (C) and sex ratio variation (D) in the after heat stress time period (AHS) in large and small populations of fighter and scrambler populations. The groups sharing the same letter are not significantly different from each other. The box shows dispersion of data between interquartile range (IQR) of first and third quartiles of data while the line in box represents the median, and whiskers show 1.5 × IQR. The black dot is the mean of the population in that treatment.

The comparison among different GAMMs of adult population size showed that the interaction between morph treatment and population type had a significant effect (*p* = 0.001, Table S1), with fighter treatment maintaining lower adult population sizes than scrambler treatment, especially in small populations (Figure 3A). The temporal trend showed that fighter populations declined initially and later recovered (*p* < 0.001), whereas scrambler populations increased initially and then plateaued (*p* < 0.001, Table S2, Figure S10). As shown in Figure 2, we also found that the replacement of scramblers with fighters in the fighter treatments was followed by an immediate decline in female population size (LM: females: *χ* ^2^₁ = 4.09, *p* = 0.04, Table S5), but this decline was not significant for males (*χ* ^2^₁ = 3.41, *p* = 0.06). We also censused tritonymphs (juveniles) to understand recruitment dynamics. Tritonymph population size was best explained by a GAMM including an interaction between morph treatment and population size (*p* < 0.001, Table S3) with fighter populations maintaining higher tritonymph numbers in large populations than small populations (*χ* ^2^₁ = 5.67, *p* = 0.01). The temporal trends showed that morph treatment did not significantly interact with time. Upon comparison of GAMMs of sex ratio, we found that adding morph treatment significantly improves model fit (*p* = 0.01, Table S7), with the fighter treatment showing a more male‐skewed sex ratio (females comprising ~30% of the population, estimated marginal mean or *emm*) than scrambler treatment (females comprising ~33% of the population, *emm*). However, this effect disappeared when the model was fitted with REML (Table S8). All the temporal trends for large and small populations for adult population size, tritonymph population size and sex ratio are presented in SI.

### 3.2 Response to Environmental Perturbations

The large fighter populations showed greater declines after heat stress (AHS) than the large scrambler populations, but the decline was similar in small populations for both the treatments (LMs: *χ* ^2^₁ = 4.36, *p* = 0.037, Figure 6, Table S9). For adult population size in the AHS period, the comparison of GAMMs showed significant effects of both morph treatment (*p* < 0.001, Figure 3B, Table S1) and population type (*p* = 0.02) with scrambler populations maintaining a higher adult population size than fighter populations. Adult population size in the fighter treatment increased during the first half of the experiment and then plateaued (*p* < 0.001), whereas scrambler populations showed a consistent increase over time (*p* < 0.001). Model comparison for tritonymphs during AHS showed a significant interaction between population size and male morph treatment (*p* < 0.001, Table S3). This interaction effect arose from a larger difference between large and small population sizes in the fighter populations than in scrambler populations (Figure S2B).

For sex ratio during the after heat stress (AHS) phase, none of the model comparisons showed significant effects of morph treatment, population type, or their interaction (Table S7). However, the coefficients table showed a significant effect of morph treatment, with scrambler treatment having less biased male‐skew in populations (Table S8).

### 3.3 Presence of Aggressive Male Morph Reduces Population Stability

**FIGURE 4:**
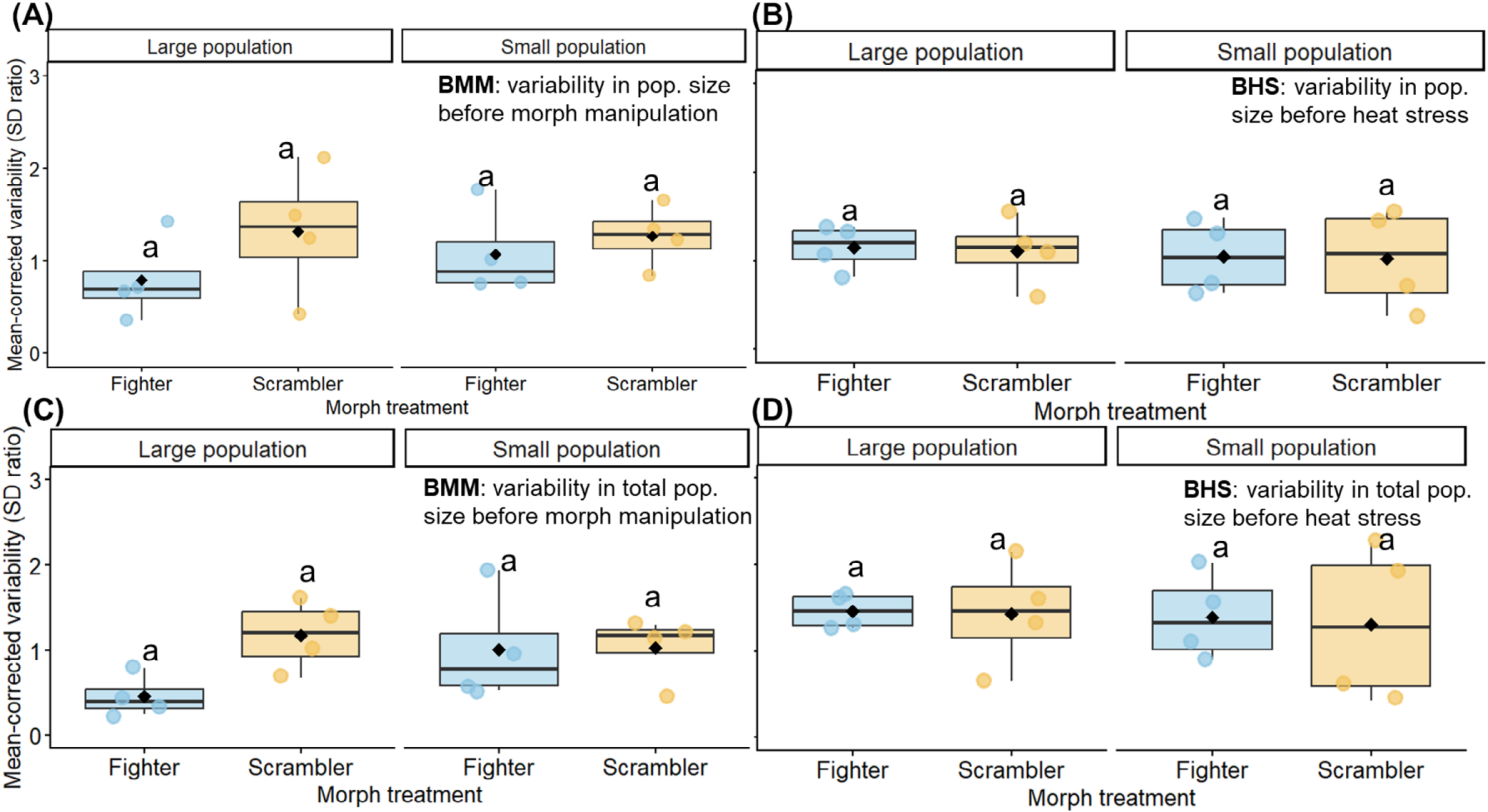
Mean‐corrected variability_adult‐population‐size_ in large and small populations of fighter and scrambler treatment (A) in the BMM (Day 38 to Day 58) (B) and BHS time period (Day 58 to Day 82). Mean‐corrected variability_total‐population‐size_ (adults + tritonymphs) in large and small populations of fighter and scrambler populations treatment (C) in the BMM (Day 38 to Day 58) (D) and BHS time period (Day 58 to Day 82). The groups sharing the same letter are not significantly different. The box shows dispersion of data between interquartile range (IQR) of first and third quartiles of data while the line in box represents the median, and whiskers show 1.5 × IQR. The black dot is the mean of the population in that treatment.

We report here results based on mean‐corrected variability as a measure of population stability (analyses of CV in Supplement text b gave similar results). When comparing stability across the before morph manipulation and before heat stress phases using mean‐corrected variability_adult‐population‐size_, the GLMMs show no effect of morph treatment, time period or their interaction (Table S16). However, mean‐corrected variability_total‐population‐size_ showed a significant morph treatment and time period interaction (*χ* ^2^₁ = 4.60, *p* = 0.03, Figure 4C,D, Table S15), suggesting that populations in the fighter treatment became more variable once morph manipulations began.

Across the before and after heat stress phases, mean‐corrected variability_adult‐population‐size_ was significantly influenced by a 3‐way interaction, with small scrambler populations showing higher instability in the AHS phase than BHS phase (*χ* ^2^₁ = 5.46, *p* = 0.02, Figure S8C,D, Table S17). For mean‐corrected variability_total‐population‐size_, there was a significant interaction between population size and morph treatment, with large fighters being most unstable (*χ* ^2^₁ = 5.10, *p* = 0.02, Table S17). Population size also interacted with time period, with small populations being more unstable in the AHS than the BHS phase (*χ* ^2^₁ = 4.21, *p* = 0.04, Table S17).

### 3.4 Population Growth and Recovery After Environmental Perturbation

**FIGURE 5:**
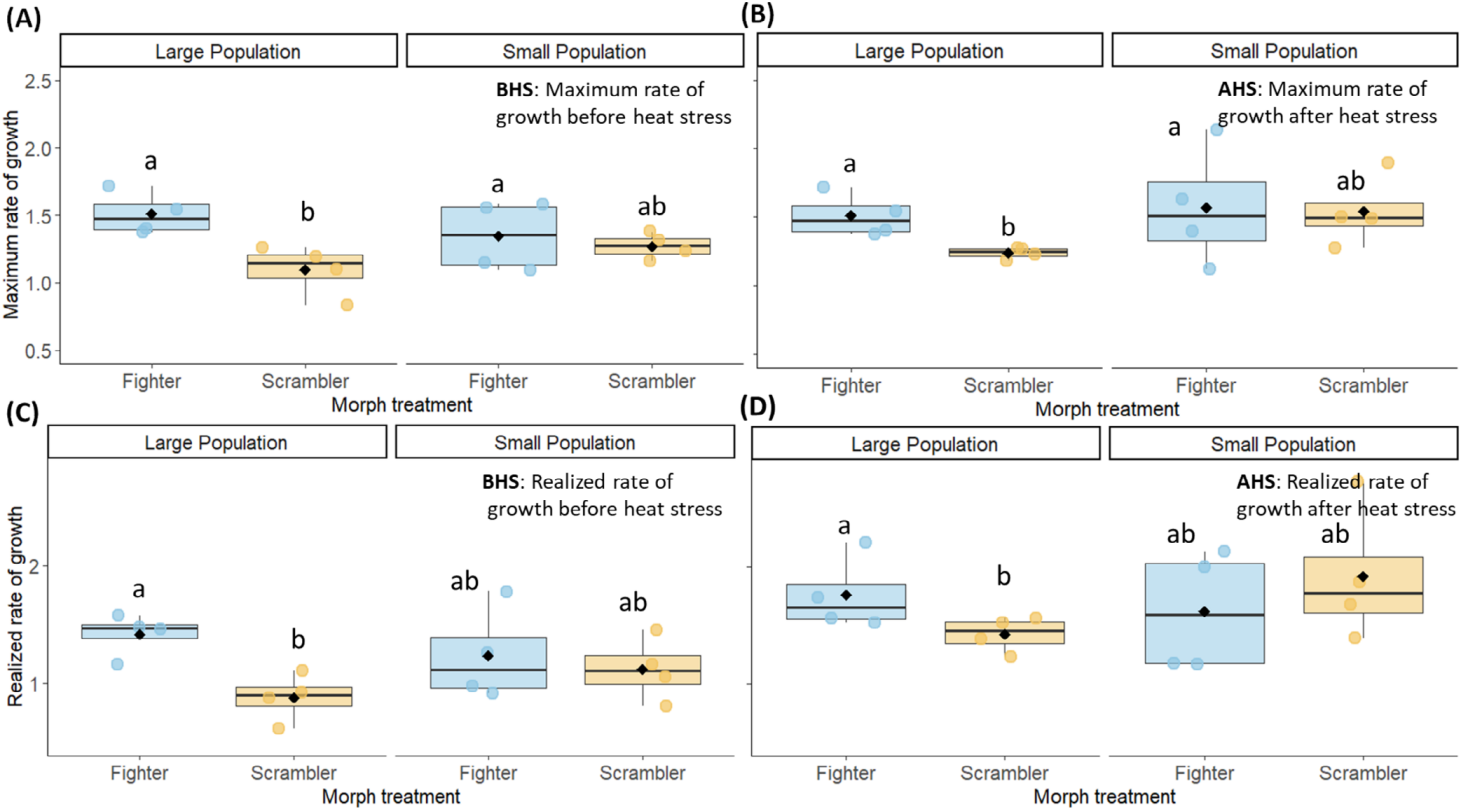
The *r*_*max*_ in large and small populations of fighter and scrambler treatment (A) in the BHS that is, before the heat stress (B) and AHS (after heat stress) time period. *r* in large and small populations of fighter and scrambler treatment (C) in the BHS that is, before the heat stress (D) and AHS (after heat stress) time period. The *r*_*max*_ and *r* were calculated using adult population size. The groups sharing the same letter are not significantly different from each other. The box shows dispersion of data between interquartile range (IQR) in first and third quartiles of data while the line in box represents the median, and whiskers show 1.5 × IQR. The black dot is the mean of the population in that treatment.

Analysis using LMs indicated a higher *r*_*max*_ (*χ* ^2^₁ = 4.01, *p* = 0.05) and *r* (*χ* ^2^₁ = 5.25, *p* = 0.02) in large fighter populations than large scramblers (Figure 5A,B). Both the *r*_*max*_ (*χ* ^2^₁ = 4.00, *p* = 0.05) and the *r* (*χ* ^2^₁ = 14.07, *p* < 0.001; Figure 5C,D, Table S19) were higher in after (AHS) than before heat stress (BHS) phase.

**FIGURE 6:**
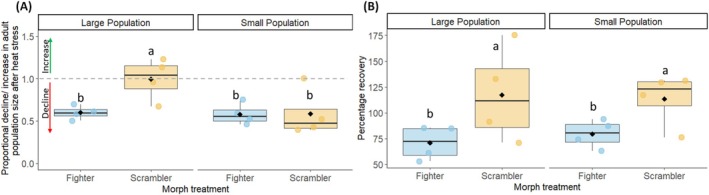
(A) Adult survivorship in large and small populations of fighter and scrambler treatment. The large scrambler population showed the lowest decline in population size. (B) Percentage recovery in large and small populations of fighter and scrambler treatment. The groups sharing the same letter are not significantly different from each other. The box shows dispersion of data between interquartile range (IQR) of first and third quartiles of data while the line in box represents the median, and whiskers show 1.5 × IQR. The black dot is the mean of the population in that treatment.

We found that scrambler treatment populations were more likely to fully recover from heat‐stress‐induced decline (*χ* ^2^₁ = 8.03, *p* = 0.004, Figure 6B) than fighter populations. This recovery did not differ between small and large populations (*χ* ^2^₁ = 0.25, *p* = 0.62, Table S21).

Fecundity was lower in small populations than large populations (*χ* ^2^₁ = 5.42, *p* = 0.02, Figure S9). Further, in the fighter treatment, females laid ~2.5 more (~100% more) eggs as compared to the females from scrambler populations (average fecundity = ~2.6 eggs) (*χ* ^2^₁ = 10.49, *p* = 0.001). The interaction between morph treatment and population size was not significant (*χ* ^2^₁ = 1.78, *p* = 0.18, Table S23).

## 4 Discussion

We explored whether the presence of a sexually selected phenotype associated with male aggression can affect the dynamics of populations, including before and after experimental heat stress. We found that the fighter populations had lower overall population sizes than the scrambler populations. Further, the fighter populations declined more sharply in response to heat stress and also showed less complete recovery than scrambler populations. Such weaker recovery possibly resulted from greater instability of fighter populations. While the effects of this fighter male phenotype were similar in both small and large populations, large fighter populations declined proportionally more following heat stress. Our results demonstrate that the ecological consequences of sexual selection and sexually selected traits can be crucial in mediating both the dynamics and recovery of populations under environmental stress, as has been predicted by models to occur in other species where sex‐specific aggression and weaponry have evolved due to intrasexual competition (Kokko and Rankin 2006; Martínez‐Ruiz and Knell 2017; Łukasiewicz et al. 2023; Porwal et al. 2025).

### 4.1 Presence of Aggressive Male Phenotype Lowers Average Population Size

Our findings suggest that pre‐copulatory sexual selection associated with male weaponry and aggression reduces population size, which was ~52% (raw arithmetic mean) lower in the populations with fighter males as compared to those with scramblers. This difference resulted from the negative effects caused by the fighters, as evidenced from the sudden decline in the population size when scramblers were replaced with fighter males (Figure 2A,B), and the increase in population size under the opposite scrambler male treatment, when fighters were replaced with scramblers. These changes in population size could be due to fighter males engaging in aggressive fights with rival males to monopolize females, which increases male mortality (Radwan 1993). However, while increased male mortality is expected to bias the sex ratio towards females, we surprisingly found that populations subjected to fighters were more male‐skewed (Figure 3) than the scrambler populations, both before and after heat stress. These male‐skewed sex ratios in fighter populations most probably arose from aggressive males reducing female survival. Such detrimental effects can arise from inter‐sexual conflict, where high mating frequency favours males but the associated harassment is costly to females (Chapman et al. 1995; Arnqvist and Rowe 2002). For example, in *S. berlesei* the longevity of females declines upon higher and multiple mating exposure to males. Although this effect was more pronounced for scrambler males in Łukasiewicz (2020), this may change under food restriction, as Łukasik (2010) reported that female mortality considerably increased in the presence of fighters, who sometimes kill and cannibalize females. These distinct pathways can promote a male‐biased sex ratio, causing changes in demographic behaviour of populations and increase extinction risk (Rankin et al. 2011). Increased female mortality associated with sexually selected male traits and behaviours is reported from many species, including elephant seals (Le Boeuf and Mesnick 1990), feral sheep (Réale et al. 1996), waterfowl (Owen and Dix 1986; Kokko et al. 1998; Adler 2010) and common lizards (Le Galliard et al. 2005; reviewed in Giery and Layman 2019). However, it is possible that the strong effects of armed males on female survivorship, as observed by us, may not be as extreme in other systems. For example, in Soay sheep where male–male competition is intense in rutting seasons (in high density years), females are affected to a lesser extent and the populations are female‐biased (Chapman et al. 2023).

### 4.2 Presence of Aggressive Male Phenotype Increased Population Instability and Vulnerability to Heat Stress

Populations in the fighter treatment showed larger fluctuations in total population size than populations in the scrambler treatment (Figure S8). Such increased instability, along with the negative effect of fighters on population size, can increase vulnerability to environmental disturbance and environmental stochasticity. This is what we found in the large populations containing fighter males, which showed a greater decline (~40% decline, raw mean) in adult population size after heat stress compared to scrambler populations (< 1% decline). In small populations, however, the decline was similar for both the morph treatments (~42% decline, raw mean). The difference between large and small populations thus appears to result from higher resilience of the large scrambler populations (Figure 2).

While heat stress resulted in comparable relative declines in small populations of both fighter and scrambler treatments, the fighter populations were already smaller before heat stress. Consequently, in small fighter populations female population size dropped to 5–6 females in two replicates. Such a decline in the number of females can make populations vulnerable to future genetic bottleneck effects and demographic stochasticity (Le Galliard et al. 2005). Overall, our findings suggest that populations with strong pre‐copulatory sexual selection associated with armed males tend to have low demographic resistance (*sensu* Capdevila et al. 2020), and can sharply decline when exposed to environmental stressors, increasing their chances of extinction. Because we replaced males in our experimental population, the bottlenecks probably did not lead to drastic declines in their genetic variability that could otherwise increase inbreeding, limiting populations' ability to adapt. While this design allowed us to focus on purely demographic effects, we may have underestimated possible detrimental effects of this sexually selected male phenotype on the chances of population recovery. This is because populations exposed to this phenotype may have lower effective population size, although they may be effective in purging genetic load (Martínez‐Ruiz and Knell 2017). Another limitation of our study is that it may overrepresent the demographic impact of such pre‐copulatory sexual selection on females, because the males brought from the stock may have been bigger and thus caused more physical harm to females during copulations and other interactions than the resident, non‐replaced males—especially so in populations with limited effective space and no refuge (Yun et al. 2018; Berger and Liljestrand‐Rönn 2024).

### 4.3 Presence of Aggressive Male Phenotype Promotes Growth Rate but Constraints Recovery After Heat Stress

The effects of fighter males on growth rate after heat stress and recovery to pre‐stress levels were complex. In large populations, both *r*_*max*_ and *r* were higher in the fighter treatment than in the scrambler treatment. This could be due to increased per‐capita food availability caused by greater population decline and a lower overall post‐perturbation population size in the fighter treatment compared to the scrambler treatment. Importantly, however, despite the growth rate advantage (*r*_*max*_: 29% higher; *r*: 38% higher, *emm*) in large populations, fighter populations did not recover fully to the population size prior to heat stress, unlike the scrambler treatment. This could be due to higher population instability and altered demographic structure of the recovering populations dominated by young recruits. We only traced our population for 12 days after the heat stress, so it cannot be excluded that the populations would fully recover in later generations when the age structure stabilizes. However, the incomplete recovery observed by us implies that recovery from stress can be delayed by strong pre‐copulatory sexual selection, thus lowering the demographic resilience to environmental stress and change. Our findings suggest that differences in initial population size may not be as important for population recovery, as both large and small populations with fighter treatment showed reduced recovery after heat stress.

### 4.4 Fecundity Increased Under Presence of Aggressive Male Morph Possibly Due to More Food

Females taken from the fighter treatment had greater fecundity which we attribute to high per‐capita food availability resulting from population declines. Previous work on beetles indicates that the males most successful in reproductive competition can negatively affect females' fecundity, possibly because they attempt longer and multiple matings which reduce female life‐time fecundity (Edvardsson and Tregenza 2005, but see Fromonteil et al. 2023). In true bugs, multiple matings can increase immediate fecundity but reduce female longevity, resulting in no net effect of multiple mating on life‐time fecundity (Backhouse et al. 2012). Considering our findings, we suggest that studies of the consequences of sexual conflict should also consider such ecological and population contexts of sexual selection, as evolved effects of sexual conflict on female fitness may be modified by common ecological feedbacks such as density‐dependent food‐limitation.

Our findings appear to support Flintham et al. (2023)'s suggestion that demographic benefits of good‐gene effects on females can be largely offset by male harm. However, while Flintham et al. found that populations declined largely due to reduced recruitment rate, we found that female mortality resulting from male aggression also affected population dynamics. Although our experiment did not address genetic effects of sexual selection, the observed demographic patterns are consistent with the idea that sexual conflict can substantially alter population dynamics, as supported by observed population dynamics outcomes caused by sexual conflict induced increases in female mortality, in several taxa, including species of conservation concern (Le Boeuf and Mesnick 1990, Réale et al. 1996, Owen and Dix 1986, Kokko et al. 1998, Adler 2010, Le Galliard et al. 2005, Brides et al. 2017; review in Giery and Layman 2019). In summary, our study demonstrates that sexual selection involving male aggression disproportionately reduces female population size, produces more unstable population dynamics which, alongside reduced female lifetime fitness, can constrain recovery following population declines. We think that such effects may be more widespread than previously considered, especially in species exhibiting sexually selected weaponry and aggression. A focus on female survival in natural populations with strong pre‐copulatory sexual selection and aggressive male mating strategies may be required to improve their conservation status. Future studies could advance the understanding of the dynamics of contemporary populations by examining the impacts of other stressors and their interaction with forces of sexual selection.

## Author Contributions

Tom C. Cameron, Jacek Radwan, Neha Pandey, Jonathan M. Parrett, Neelam Porwal, and Robert J. Knell conceived the ideas and designed methodology; Neha Pandey, Neelam Porwal, and Jonathan M. Parrett collected the data; Neha Pandey and Robert J. Knell analysed the data with support from Jonathan M. Parrett; and Neha Pandey, Tom C. Cameron, and Jacek Radwan led the writing of the manuscript with contributions from Jonathan M. Parrett, Robert J. Knell. All authors contributed critically to the drafts and gave final approval for publication.

## Funding

This work was supported by Narodowe Centrum Nauki (UMO‐2020/39/B/NZ8/00152/4).

## Supporting information


**Figure S1:** Number of emerging fighter males in large and small populations of fighter and scrambler treatment prior to male morph manipulation. The error bars around the mean are standard errors.
**Figure S2:** Number of tritonymphs in the time period (A) BHS (before heat stress) and (B) AHS (after heat stress) in large and small populations of fighter and scrambler treatment. The groups sharing the same letter are not significantly different. The box shows dispersion of data between interquartile range (IQR) of first and third quartiles of data while the line in box represents the median, and whiskers show 1.5 × IQR. The black dot is the mean of the population in that treatment.
**Figure S3:** Tritonymph population size time series in (A) large and (B) small populations of the scrambler and fighter treatments. The dark blue colour shows the mean tritonymph population size in the fighter male‐morph treatment, and the dark orange colour represents the mean tritonymph population size in the scrambler male‐morph population, while all the lighter colours show tritonymph population size in the respective replicate populations. The fighter and scrambler male‐morph treatments were established by replacing male morphs from the stock populations from day 58 from the experiment setup. On day 82 from setup, the populations were given a heat stress for 90 h at 40°C. Note: Tritonymph census were done on every alternate day except for the period between 62 day and 78 day when census was done every 4 days.
**Figure S4:** (A) Decline in male survivorship (day 60) after male morphs were replaced. (B) Decline in female survivorship (day 60) after male morphs were replaced. The groups sharing the same letter are not significantly different. The box shows dispersion of data between interquartile range (IQR) of first and third quartiles of data while the line in box represents the median, and whiskers show 1.5 × IQR. The black dot is the mean of the population in that treatment.
**Figure S5:** Sex ratio time series in (A) large and (B) small populations of scrambler and fighter treatments. The dark blue colour shows the mean sex ratio in the fighter male‐morph treatment, and the dark orange colour represents the mean sex ratio in the scrambler male‐morph population, while all the lighter colours show sex ratio in the respective replicate populations. The fighter and scrambler male‐morph treatments were established by replacing male morphs from the stock populations from day 58 from the experiment setup. On Day 82 from setup, the populations were given a heat wave for 90 h at 40°C. Note: Population census were done on every alternate day except for the period between 62 day and 78 day when census was done every 4 days.
**Figure S6:** CV_adult‐population‐size_ in large and small populations of fighter male‐morph and scrambler male‐morph treatment (A) in the BMM (Day 38 to Day 58) (B) and BHS time period. CV_total‐population‐size_ (adults + tritonymphs) in large and small populations of fighter and scrambler populations treatment (C) in the BMM (Day 38 to Day 58) (D) and BHS time period. The groups sharing the same letter are not significantly different, but † indicate a marginal trend. The box shows dispersion of data between interquartile range (IQR) of first and third quartiles of data while the line in box represents the median, and whiskers show 1.5 × IQR. The black dot is the mean of the population in that treatment.
**Figure S7:** CV_adult‐population‐size_ in large and small populations of fighter male‐morph and scrambler male‐morph treatment (A) in the BHS (B) and AHS time period. CV_total‐population‐size_ (adults + tritonymphs) in large and small populations of fighter and scrambler populations treatment (C) in the BHS (D) and AHS time period. The groups sharing the same letter are not significantly different. The box shows dispersion of data between interquartile range (IQR) of first and third quartiles of data while the line in box represents the median, and whiskers show 1.5 × IQR. The black dot is the mean of the population in that treatment.
**Figure S8:** Mean‐corrected variability_adult‐population‐size_ in large and small populations of fighter and scrambler treatment (A) in the BHS that is, before the heat stress (B) and AHS time period, and the Mean‐corrected variability_adult‐population‐size_ was calculated using adult population size. Mean‐corrected variability_total‐population‐adult‐size_ (adults + tritonymphs) in large and small populations of fighter and scrambler populations treatment (C) in the BHS that is, before the heat stress (D) and AHS time period, and the Mean‐corrected variability_adult‐population‐size_ was calculated using total adult population size. The groups sharing the same letter are not significantly different. The box shows dispersion of data between interquartile range (IQR) of first and third quartiles of data while the line in box represents the median, and whiskers show 1.5 × IQR. The black dot is the mean of the population in that treatment.
**Figure S9:** Fecundity per female in large and small populations of fighter male‐morph and scrambler male‐morph treatment after the population dynamics experiment. The females were allowed to lay eggs for 16 h (*n* = 8 per treatment combination). The groups sharing the same letter are not significantly different. The box shows dispersion of data between interquartile range (IQR) of first and third quartiles of data while the line in box represents the median, and whiskers show 1.5 × IQR. The black dot is the mean of the population in that treatment.
**Figure S10:** Temporal dynamics in adult population size across male morph treatments (fighter, scrambler) and population sizes (large, small) in the before‐heat‐stress (BHS) period.
**Figure S11:** Temporal dynamics in adult population size across male morph treatments (fighter, scrambler) and population sizes (large, small) in the after‐heat‐stress (AHS) period.
**Figure S12:** Temporal dynamics in the tritonymph population across population sizes (large, small) in the before‐heat‐stress (BHS) period.
**Figure S13:** Temporal dynamics in the tritonymph population across population sizes (large, small) in the after‐heat‐stress (AHS) period.
**Figure S14:** Temporal dynamics in the sex ration variation across population sizes (large, small) in the before‐heat‐stress (BHS) period.
**Table S1:** Model comparison results for adult population size in the BHS and AHS time periods using nested generalized additive mixed models (GAMMs).
**Table S2:** Coefficients from the best‐fitting generalized additive mixed‐effects model (GAMM) for adult population size in the BHS and AHS time periods. The tables below present the estimated parametric coefficients and smooth terms from the selected models for BHS and AHS time period.
**Table S3:** Model comparison *F* test results from model comparisons of generalized additive mixed models (GAMMs) for tritonymph population size in the BHS and AHS time periods.
**Table S4:** Coefficients from the best‐fitting generalized additive mixed‐effects model (GAMM) for tritonymph population size in the BHS and AHS time periods. The tables below present the estimated parametric coefficients and smooth terms from the selected models for BHS and AHS time period.
**Table S5:** Results from stepwise removal of fixed effects in linear mixed effects model for male and female survivorship once the wrong morphs were replaced with right morphs.
**Table S6:** Coefficients from the linear mixed‐effects model for male survivorship after morph replacement, and female survivorship after replacement.
**Table S7:** Results of *F* tests comparing nested generalized additive mixed models (GAMMs) for log‐transformed sex ratio in the BHS and AHS time periods.
**Table S8:** Coefficients from the best‐fitting generalized additive mixed‐effects model (GAMM) for sex ratio in the BHS and AHS time periods. The tables below present the estimated parametric coefficients and smooth terms from the selected models for BHS and AHS time period.
**Table S9:** Results from stepwise removal of fixed effects in linear mixed effects model for decline in adult population size after heat stress.
**Table S10:** Coefficients from the linear mixed‐effects model for decline in population size after heat stress.
**Table S11:** Results from stepwise removal of fixed effects in generalized mixed effects model using gamma error structure for CV_adult‐population‐size_ and CV_total‐population‐size_ before and after morph manipulation.
**Table S12:** Coefficients from the generalized mixed‐effects model for CV_adult‐population‐size_ and CV_total‐population‐size_ before and after morph manipulation. For CV_adult‐population‐size_, the variance explained by random effect, that is, population identity was 0.044. For CV_total‐population‐size_, the variance explained by population identity was 0.217.
**Table S13:** Results from stepwise removal of fixed effects in generalized mixed effects model using gamma error structure for CV_adult‐population‐size_ and CV_total‐population‐size_ before and after heat stress.
**Table S14:** Coefficients from the generalized mixed‐effects model for CV_adult‐population‐size_ and CV_total‐population‐size_. For CV_adult‐population‐size_, the variance explained by random effect, that is, population identity was 0.841. For CV_total‐population‐size_, the variance explained by population identity was < 0.001.
**Table S15:** Results from stepwise removal of fixed effects in generalized mixed effects model using gaussian error structure for Mean‐corrected variability_adult‐population‐size_ and Mean‐corrected variability_total‐population‐size_ before and after morph manipulation.
**Table S16:** Coefficients from the generalized mixed‐effects model for Mean‐corrected variability_adult‐population‐size_ and Mean‐corrected variability_total‐population‐size_ before and after morph manipulation. For Mean‐corrected variability_adult‐population‐size_, the variance explained by random effect, that is, population identity was 0.0012. For Mean‐corrected variability_total‐population‐size_, the variance explained by population identity was 0.006.
**Table S17:** Results from stepwise removal of fixed effects in generalized mixed effects model using gamma error structure for Mean‐corrected variability_adult‐population‐size_ and Mean‐corrected variability_total‐population‐size_ before and after heat stress.
**Table S18:** Coefficients from the generalized mixed‐effects model for Mean‐corrected variability_adult‐population‐size_ and Mean‐corrected variability_total‐population‐size_ before and after heat stress. For Mean‐corrected variability_adult‐population‐size_, the variance explained by random effect, that is, population identity was < 0.001. For Mean‐corrected variability_total‐population‐size_, the variance explained by population identity was < 0.001.
**Table S19:** Results from stepwise removal of fixed effects in linear mixed‐effects for maximum and realized rate of growth before and after heat stress.
**Table S20:** Coefficients from the linear mixed‐effects model for maximum and realized rate of growth. For maximum rate of growth, the variance explained by random effect, that is, population identity was < 0.001. For realized rate of growth, the variance explained by population identity was < 0.001.
**Table S21:** Results from stepwise removal of fixed effects in linear mixed‐effects model for percentage recovery of populations after heat stress.
**Table S22:** Coefficients from the linear mixed‐effects model for percentage recovery after heat stress.
**Table S23:** Results from stepwise removal of fixed effects in generalized mixed‐effects model with negative binomial error structure for fecundity.
**Table S24:** Coefficients from the generalized mixed‐effects model for fecundity. For fecundity, the variance explained by random effect, that is, population identity was < 0.001.

## Data Availability

The r‐codes and data supporting the findings are archived in a public repository; DOI: https://zenodo.org/records/18482842.

## References

[ele70377-bib-0001] Adler, M. 2010. “Sexual Conflict in Waterfowl: Why Do Females Resist Extrapair Copulations?” Behavioral Ecology 21: 182–192.

[ele70377-bib-0002] Andersson, M. 1994. Sexual Selection. Princeton University Press.

[ele70377-bib-0003] Arnqvist, G. , and L. Rowe . 2002. “Antagonistic Coevolution Between the Sexes in a Group of Insects.” Nature 415: 787–789. 11845208 10.1038/415787a

[ele70377-bib-0004] Backhouse, A. , S. M. Sait , and T. C. Cameron . 2012. “Multiple Mating in the Traumatically Inseminating Warehouse Pirate Bug, *Xylocoris flavipes* : Effects on Fecundity and Longevity.” Biology Letters 8: 706–709. 22573833 10.1098/rsbl.2012.0091 PMC3440960

[ele70377-bib-0005] Beckerman, A. P. , T. G. Benton , C. T. Lapsley , and N. Koesters . 2003. “Talkin Bout My Generation: Environmental Variability and Cohort Effects.” American Naturalist 162: 754–767. 10.1086/381056 14737713

[ele70377-bib-0006] Benton, T. G. , and A. P. Beckerman . 2005. “Population Dynamics in a Noisy World: Lessons From a Mite Experimental System.” Advances in Ecological Research 37: 143–181.

[ele70377-bib-0007] Berger, D. , and J. Liljestrand‐Rönn . 2024. “Environmental Complexity Mitigates the Demographic Impact of Sexual Selection.” Ecology Letters 27: 1–12. 10.1111/ele.14355 38225825

[ele70377-bib-0008] Brides, K. , K. A. Wood , R. D. Hearn , and T. P. M. Fijen . 2017. “Changes in the Sex Ratio of the Common Pochard *Aythya ferina* in Europe and North Africa.” Widlfowl 67: 100–112.

[ele70377-bib-0009] Bro‐Jørgensen, J. 2014. “Will Their Armaments Be Their Downfall? Large Horn Size Increases Extinction Risks in Bovids.” Animal Conservation 17: 80–87.

[ele70377-bib-0010] Brooks, M. , B. Bolker , K. Kristensen , et al. 2024. Generalized Linear Mixed Models Using Template Model Builder. CRAN: Contributed Packages.

[ele70377-bib-0011] Brooks, M. E. , K. Kristensen , K. J. Van Benthem , et al. 2017. glmmTMB Balances Speed and Flexibility Among Packages for Zero‐Inflated Generalized Linear Mixed Modeling. The R Journal.

[ele70377-bib-0012] Cameron, T. C. , and T. G. Benton . 2004. “Stage‐Structured Harvesting and Its Effects: An Empirical Investigation Using Soil Mites.” Journal of Animal Ecology 73: 996–1006.

[ele70377-bib-0013] Candolin, U. , and J. Heuschele . 2008. “Is Sexual Selection Beneficial During Adaptation to Environmental Change?” Trends in Ecology & Evolution 23: 446–452. 18582989 10.1016/j.tree.2008.04.008

[ele70377-bib-0014] Capdevila, P. , I. Stott , M. Beger , and R. Salguero‐Gómez . 2020. “Towards a Comparative Framework of Demographic Resilience.” Trends in Ecology & Evolution 35: 776–786. 32482368 10.1016/j.tree.2020.05.001

[ele70377-bib-0015] Chapman, E. G. , J. Pilkington , and J. M. Pemberton . 2023. “Correlates of Early Reproduction and Apparent Fitness Consequences in Male Soay Sheep.” Ecology and Evolution 13, no. 5: e10058. 37168987 10.1002/ece3.10058 PMC10164647

[ele70377-bib-0016] Chapman, T. , L. F. Liddle , J. M. Kalb , M. F. Wolfner , and L. Partridge . 1995. “Cost of Mating in *Drosophila melanogaster* Females Is Mediated by Male Accessory Gland Products.” Nature 373: 241–244. 7816137 10.1038/373241a0

[ele70377-bib-0017] Clutton‐Brock, T. H. , K. E. Rose , and F. E. Guinness . 1997. “Density‐Related Changes in Sexual Selection in Red Deer.” Proceedings of the Biological Sciences 264: 1509–1516. 9364790 10.1098/rspb.1997.0209 PMC1688709

[ele70377-bib-0018] Cunningham, S. J. , J. L. Gardner , and R. O. Martin . 2021. “Opportunity Costs and the Response of Birds and Mammals to Climate Warming.” Frontiers in Ecology and the Environment 19, no. 5: 300–307.

[ele70377-bib-0019] Darwin, C. 1871. The Descent of Man, and Selection in Relation to Sex. John Murray.

[ele70377-bib-0020] Dugand, R. J. , J. L. Tomkins , and W. J. Kennington . 2019. “Molecular Evidence Supports a Genic Capture Resolution of the Lek Paradox.” Nature Communications 10: 1359. 10.1038/s41467-019-09371-y PMC6433924 30911052

[ele70377-bib-0021] Eberhart‐Phillips, L. J. , C. Küpper , T. E. X. Miller , et al. 2017. “Sex‐Specific Early Survival Drives Adult Sex Ratio Bias in Snowy Plovers and Impacts Mating System and Population Growth.” Proceedings of the National Academy of Sciences, USA 114: E5474–E5481. 10.1073/pnas.1620043114 PMC5502594 28634289

[ele70377-bib-0022] Edvardsson, M. , and T. Tregenza . 2005. “Why Do Male *Callosobruchus maculatus* Harm Their Mates?” Behavioral Ecology 16: 788–793.

[ele70377-bib-0023] Emlen, D. J. 2008. “The Evolution of Animal Weapons.” Annual Review of Ecology, Evolution, and Systematics 39: 387–413.

[ele70377-bib-0024] Fisher, R. A. 1930. The Genetical Theory of Natural Selection. Clarendon Press.

[ele70377-bib-0025] Flintham, E. O. , V. Savolainen , and C. Mullon . 2023. “Male Harm Offsets the Demographic Benefits of Good Genes.” Proceedings of National Academy of Sciences, USA 120: 1–9. 10.1073/pnas.2211668120 PMC10013744 36862690

[ele70377-bib-0026] Fromonteil, S. , L. Marie‐Orleach , L. Winkler , and T. Janicke . 2023. “Sexual Selection in Females and Evolution of Polyandry.” PLoS Biology 21, no. 1: e3001916. 36626380 10.1371/journal.pbio.3001916 PMC9831318

[ele70377-bib-0027] Fuller, A. , D. Mitchell , S. K. Maloney , and R. S. Hetem . 2016. “Towards a Mechanistic Understanding of the Responses of Large Terrestrial Mammals to Heat and Aridity Associated With Climate Change.” Climate Change Responses 3: 10.

[ele70377-bib-0028] Giery, S. T. , and C. G. Layman . 2019. “Ecological Consequences of Sexually Selected Traits: An Eco‐Evolutionary Perspective.” Quarterly Review of Biology 94: 29–74.

[ele70377-bib-0029] Hartig, F. 2024. DHARMa: Residual Diagnostics for Hierarchical (Multi‐Level/Mixed) Regression Models. CRAN: Contributed Packages.

[ele70377-bib-0030] Hosken, D. J. , J. L. Fitzpatrick , T. Pizzari , and D. J. Hodgson . 2025. “On Sperm Length Mean–Variance Relationships.” Journal of Evolutionary Biology 38: 1548–1555. 40928466 10.1093/jeb/voaf103

[ele70377-bib-0031] Jacomb, F. , J. Marsh , and L. Holman . 2016. “Sexual Selection Expediates the Evolution of Pesticide Resistance.” Evolution 70: 2746–2751. 27677862 10.1111/evo.13074

[ele70377-bib-0032] Kokko, H. , and R. Brooks . 2003. “Sexy to Die for? Sexual Selection and the Risk of Extinction.” Annales Zoologici Fennici 40: 207–219.

[ele70377-bib-0033] Kokko, H. , H. Pöysa , J. Lindström , and E. Ranta . 1998. “Assessing the Impact of Spring Hunting on Waterfowl Populations.” Annales Zoologici Fennici 35: 195–204.

[ele70377-bib-0034] Kokko, H. , and D. J. Rankin . 2006. “Lonely Hearts or Sex in the City? Density‐Dependent Effects in Mating Systems.” Philosophical Transactions of the Royal Society, B: Biological Sciences 361, no. 1466: 319–334. 10.1098/rstb.2005.1784 PMC1569612 16612890

[ele70377-bib-0035] Le Boeuf, B. J. , and S. Mesnick . 1990. “Sexual Behavior of Male Northern Elephant Seals: 1.” Lethal Injuries to Adult Females. Behaviour 116: 143–162.

[ele70377-bib-0036] Le Galliard, J. F. , J. Cote , and P. S. Fitze . 2008. “Lifetime and Intergenerational Fitness Consequences of Harmful Male Interactions for Female Lizards.” Ecology, USA 89: 56–64. 10.1890/06-2076.1 18376547

[ele70377-bib-0037] Le Galliard, J. F. , P. S. Fitze , R. Ferrière , and J. Clobert . 2005. “Sex Ratio Bias, Male Aggression, and Population Collapse in Lizards.” Proceedings of the National Academy of Sciences, USA 102: 18231–18236. 10.1073/pnas.0505172102 PMC1312374 16322105

[ele70377-bib-0038] Lindström, J. , and H. Kokko . 2002. “Cohort Effect and Population Dynamics.” Ecology Letters 5: 338–344.

[ele70377-bib-0040] Lorch, P. D. , S. Proulx , L. Rowe , and T. Day . 2003. “Condition‐Dependent Sexual Selection Can Accelerate Adaptation.” Evolutionary Ecology Research 5: 867–881.

[ele70377-bib-0041] Łukasiewicz, A. 2020. “Juvenile Diet Quality and Intensity of Sexual Conflict in the Mite *Sancassania berlesei* .” BMC Evolutionary Biology 20: 1–11. 32164531 10.1186/s12862-020-1599-5 PMC7069193

[ele70377-bib-0042] Łukasiewicz, A. , N. Porwal , M. Niśkiewicz , J. M. Parrett , and J. Radwan . 2023. “Sexually Selected Male Weapon Increases the Risk of Population Extinction Under Environmental Change: An Experimental Evidence.” Evolution 77: 2291–2300. 37503764 10.1093/evolut/qpad139

[ele70377-bib-0043] Łukasik, P. 2010. “Trophic Dimorphism in Alternative Male Reproductive Morphs of the Acarid Mite *Sancassania berlesei* .” Behavioral Ecology 21: 270–274.

[ele70377-bib-0044] Lumley, A. J. , Ł. Michalczyk , J. J. N. Kitson , et al. 2015. “Sexual Selection Protects Against Extinction.” Nature 522: 470–473. 25985178 10.1038/nature14419

[ele70377-bib-0045] Marler, C. , and M. Moore . 1988. “Evolutionary Costs of Aggression Revealed by Testosterone Manipulations in Free‐Living Male Lizards.” Behavioral Ecology and Sociobiology 23: 21–26.

[ele70377-bib-0046] Martínez‐Ruiz, C. , and R. J. Knell . 2017. “Sexual Selection Can Both Increase and Decrease Extinction Probability; Reconciling Demographic and Evolutionary Factors.” Journal of Animal Ecology 86: 117–127. 27861841 10.1111/1365-2656.12601

[ele70377-bib-0047] Martins, M. J. F. , T. M. Puckett , R. Lockwood , J. P. Swaddle , and G. Hunt . 2018. “High Male Sexual Investment as a Driver of Extinction in Fossil Ostracods.” Nature 556: 366–369. 29643505 10.1038/s41586-018-0020-7

[ele70377-bib-0049] Owen, M. , and M. Dix . 1986. “Sex Ratios in Some Common British Wintering Ducks.” Wild 37: 104–112.

[ele70377-bib-0050] Ozgul, A. , T. Coulson , A. Reynolds , T. C. Cameron , and T. G. Benton . 2012. “Population Responses to Perturbations: The Importance of Trait‐Based Analysis Illustrated Through a Microcosm Experiment.” American Naturalist 179: 582–594. 10.1086/664999 22504541

[ele70377-bib-0051] Parrett, J. M. , S. Chmielewski , E. Aydogdu , et al. 2022. “Genomic Evidence That a Sexually Selected Trait Captures Genome‐Wide Variation and Facilitates the Purging of Genetic Load.” Nature Ecology & Evolution 6: 1330–1342. 35851852 10.1038/s41559-022-01816-w

[ele70377-bib-0052] Parrett, J. M. , and R. J. Knell . 2018. “The Effect of Sexual Selection on Adaptation and Extinction Under Increasing Temperatures.” Proceedings of the Royal Society B: Biological Sciences 285: 285. 10.1098/rspb.2018.0303 PMC5936732 29669902

[ele70377-bib-0053] Plesnar‐Bielak, A. , A. M. Skrzynecka , Z. M. Prokop , and J. Radwan . 2012. “Mating System Affects Population Performance and Extinction Risk Under Environmental Challenge.” Proceedings of the Royal Society B‐Biological Sciences 279: 4661–4667. 10.1098/rspb.2012.1867 PMC3479737 22977151

[ele70377-bib-0054] Porwal, N. , J. M. Parrett , A. Szubert‐Kruszyńska , et al. 2025. “Fighting Through the Heat: How Male Aggression Influences Demography Under Recurrent Heatwaves.” Ecology and Evolution 15: e72034. 40978220 10.1002/ece3.72034 PMC12446712

[ele70377-bib-0055] R Core Team . 2025. R: A Language and Environment for Statistical Computing. R Foundation for Statistical Computing.

[ele70377-bib-0056] Radwan, J. 1993. “The Adaptive Significance of Male Polymorphism in the Acarid Mite *Caloglyphus berlesei* .” Behavioral Ecology and Sociobiology 33: 201–208.

[ele70377-bib-0057] Radwan, J. 1995. “Male Morph Determination in Two Species of Acarid Mites.” Heredity 74: 669–673.

[ele70377-bib-0058] Radwan, J. , and M. Rysińska . 1999. “Effect of Mating Frequency on Female Fitness in *Caloglyphus berlesei* (Astigmata: Acaridae).” Experimental & Applied Acarology 23, no. 5: 399–409.

[ele70377-bib-0060] Radwan, J. , J. Unrug , and J. L. Tomkins . 2002. “Status‐Dependence and Morphological Trade‐Offs in the Expression of a Sexually Selected Character in the Mite, *Sancassania berlesei* .” Journal of Evolutionary Biology 15: 744–752.

[ele70377-bib-0061] Rankin, D. J. , U. Dieckmann , and H. Kokko . 2011. “Sexual Conflict and the Tragedy of Commons.” American Naturalist 177: 780–791. 10.1086/659947 21597254

[ele70377-bib-0062] Rankin, D. J. , and H. Kokko . 2007. “Do Males Matter? The Role of Males in Population Dynamics.” Oikos 116: 335–348.

[ele70377-bib-0063] Réale, D. , P. Boussès , and J. L. Chapuis . 1996. “Female‐Biased Mortality Induced by Male Sexual Harassment in a Feral Sheep Population.” Canadian Journal of Zoology 74: 1812–1818.

[ele70377-bib-0064] Rowe, L. , and H. D. Rundle . 2021. “The Alignment of Natural and Sexual Selection.” Annual Review of Ecology, Evolution, and Systematics 52: 499–517.

[ele70377-bib-0065] Spooner, F. E. B. , G. P. Richard , and R. Freeman . 2018. “Rapid Warming Is Associated With Population Decline Among Terrestrial Birds and Mammals Globally.” Global Change Biology 24, no. 10: 4521–4531. 30033551 10.1111/gcb.14361

[ele70377-bib-0069] Tuschhoff, E. , and J. J. Wiens . 2023. “Evolution of Sexually Selected Traits Across Animals.” Frontiers in Ecology and Evolution 11: 1–12.38516293

[ele70377-bib-0070] Vasilieva, N. , and A. Tchabovsky . 2015. “A Shortage of Males Causes Female Reproductive Failure in Yellow Ground Squirrels.” Science Advances 1: e1500401. 26601284 10.1126/sciadv.1500401 PMC4646798

[ele70377-bib-0071] Whitlock, M. C. 2000. “Fixation of New Alleles and the Extinction of Small Populations: Drift Load, Beneficial Alleles, and Sexual Selection.” Evolution 54, no. 6: 1855–1861. 11209765 10.1111/j.0014-3820.2000.tb01232.x

[ele70377-bib-0072] Whitlock, M. C. , and A. Agrawal . 2009. “Purging the Genome With Sexual Selection: Reducing Mutation Load Through Selection on Males.” Evolution 63, no. 3: 569–582. 19154364 10.1111/j.1558-5646.2008.00558.x

[ele70377-bib-0073] Wood, S. , and M. S. Wood . 2015. “Package ‘mgcv’.” R Package Version 1, no. 29: 729.

[ele70377-bib-0074] Yun, L. , P. J. Chen , K. E. Kwok , C. S. Angell , H. D. Rundle , and A. F. Agrawal . 2018. “Competition for Mates and the Improvement of Nonsexual Fitness.” Proceedings of the National Academy of Sciences 115, no. 26: 6762–6767. 10.1073/pnas.1805435115 PMC6042133 29891650

[ele70377-bib-0075] Zuur, A. , E. N. Ieno , and N. Walker . 2009. Mixed Effects Models and Extensions in Ecology With R (Statistics for Biology and Health) (2009 Edition). Springer.

