# Supplementary material for: Sexual Selection Associated With an Aggressive Male Phenotype Reduces Population Size and Hinders Population Recovery After Heat Stress
Source: Ecol Lett. 2026 Apr 17;29(4):e70377. doi: 10.1111/ele.70377 (PMC13089945; doi:10.1111/ele.70377)
Supplement: Supplementary file 1 — Figure S1: Number of emerging fighter males in large and small populations of fighter and scrambler treatment prior to male morph manipulation. The error bars around the mean are standard errors. Figure S2: Number of tritonymphs in the time period (A) BHS (before heat stress) and (B) AHS (after heat stress) in large and small populations of fighter and scrambler treatment. The groups sharing the same letter are not significantly different. The box shows dispersion of data between interquartile range (IQR) of first and third quartiles of data while the line in box represents the median, and whiskers show 1.5 × IQR. The black dot is the mean of the population in that treatment. Figure S3: Tritonymph population size time series in (A) large and (B) small populations of the scrambler and fighter treatments. The dark blue colour shows the mean tritonymph population size in the fighter male‐morph treatment, and the dark orange colour represents the mean tritonymph population size in the scrambler male‐morph population, while all the lighter colours show tritonymph population size in the respective replicate populations. The fighter and scrambler male‐morph treatments were established by replacing male morphs from the stock populations from day 58 from the experiment setup. On day 82 from setup, the populations were given a heat stress for 90 h at 40°C. Note: Tritonymph census were done on every alternate day except for the period between 62 day and 78 day when census was done every 4 days. Figure S4: (A) Decline in male survivorship (day 60) after male morphs were replaced. (B) Decline in female survivorship (day 60) after male morphs were replaced. The groups sharing the same letter are not significantly different. The box shows dispersion of data between interquartile range (IQR) of first and third quartiles of data while the line in box represents the median, and whiskers show 1.5 × IQR. The black dot is the mean of the population in that treatment. Figure S5: Sex rati [file ELE-29-0-s001.docx]

**SUPPLEMENTARY MATERIAL:**

**Supplement text a: Methods**

**Stock populations:** We performed a multigenerational experiment on soil mites (*Sancassania berlesei*). The stock population were collected in year 2022 from a poultry farm in Dluga Goslina, Wielkapolska, Poznan, Poland, and has been maintained in large population sizes (>1000 individuals) with overlapping generations. The stock population is maintained in plastic vials of ~2 cm diameter and have a ~5mm hole at top which is plugged with non-absorbent cotton to enable gas exchange, while the lower base is cemented with charcoal-mixed plaster-of-Paris. Populations are maintained at high humidity (>90%) with the help of water soaked paper towels at a constant temperature of 23 ℃ in dark incubators. The egg-to-adult development time in this species with ample food and at 23℃ can be around 12 days. The sex ratio and the proportion of the fighter and scrambler male morphs are not controlled in the stock population, but fighter male expression is expected to be lower at high population density (Radwan 1993). The stock population is fed with dry yeast twice a week.

Average weight of yeast balls: 0.000242 gram per ball, std.dev: 0.000025

**Fecundity experiment**: To understand the role of fecundity in the after-heat stress period, we performed a fecundity assay on the females from the replicate populations of the scrambler and fighter treatments. At the end of the population dynamics experiment, we took eight females from each (age not uniform) replicate population i.e., 128 females in total. We provided them with one ball of standard yeast in glass vials (~1cm diameter) and allowed them to lay eggs for 16 hours, after which we counted the eggs immediately. Females taken for the fecundity assay most likely mated with fighter males if they came from the fighter treatment, and with scrambler males if they came from the scrambler treatment. During the egg-laying period, females were not housed with any males. We assumed that all the females taken from each experimental vial were mated as they were maintained in an overlapping-generation cycle, thus providing a real egg-laying rate in the population dynamics experiment. Females of acarid mites will not lay fully developed eggs unless they are fertilized (Oliver 1971), and over 99% of eggs would hatch on average (Radwan and Siva-Jothy 1996), so egg count is a reliable measure of fecundity.

**Pilot Study: Adult population size at different quantities of food from day 32 of the set-up** For small populations 5, 7, 9 balls were used, and for medium populations 25, 28, 31 balls were used. The food was given every ~56 hours.

**Population dynamics experiment:** We maintained eight populations each in the fighter and scrambler treatment where pre-copulatory sexual selection is expected to be different. We observed the population dynamics in these two treatments under two levels of population size: small and large (n=4 each treatment combination). To each treatment level, we induced stress in the form of a heat stress event and compared pre-stress and post-stress population dynamics and recovery (Fig. 2).

**Replacement rule:** We followed this rule from 58^th^ day when the morph replacement began.

| \| **Morph treatment** \| **Population size** \| **Scenario** \| **Replacement rule** \| \| --- \| --- \| --- \| --- \| |
| --- | --- | --- | --- | --- |
| \| Fighter treatment (Rep. 1) \| 100 (large) \| 4 scramblers emerge \| Replace 4 scramblers with 4 freshly emerged fighters from stock \| \| --- \| --- \| --- \| --- \| |
| \| Scrambler treatment (Rep. 1) \| 50 (large) \| 2 fighters emerge \| Replace 2 Fighters with 2 Scramblers from stock \| \| --- \| --- \| --- \| --- \| |

When no fighters emerged in the scrambler treatment, the freshly emerged scramblers were replaced with freshly emerged scramblers from the stock population, in the same proportion as the scramblers were replaced in the fighter treatment.

**The coefficient of variation (CV) in population size (both adult and total population):**

We quantified population stability by calculating the coefficient of variation (CV) in population size across time, with lower CV signifying greater population stability, as:

$CV=$ $(standard deviation in population size)/(mean population size)$

For the BMM treatment, CV in population size was analysed using data points from 34-38, 38-42, 42-46, 46-50, 50-54, 54-58, whereas for the BHS treatment, data points from 58-62, 62-66, 66-70, 70-74, 74-78 and 78-82 were used.

For the BHS treatment, CV in population size was analyzed using data points from 70–74, 74–78, and 78–82, whereas for the AHS treatment, data points from 86–90, 90–94 and 94–98 were used.

However, since CV did not meet the assumption of a linear increase in standard deviation with the mean for one of the datasets, we instead rely on mean-corrected variability in the main text to infer any effects on stability.

**Supplement text b: Results for temporal trends**

Adults:

Prior to heat stress, the large populations show a consistent decline in adult population size (*P*=0.01), while the small did not show any temporal change (*P*=0.01, Fig. S10).

After heat stress, large populations showed stable population size for first half of the time series, and then it started to decline (*P*<0.001). The small populations did not show any temporal change (*P*=0.84, Fig.S11).

Tritonymphs:

Before heat stress, tritonymphs in large populations kept on increasing and plateaued in the end (*P*<0.001), whereas tritonymphs in small populations showed a consistent tritonymph count over time (*P*=0.26, Fig. S12). Morph treatment did not significantly interact with the smoother.

Both large (*P*<0.001), and small populations (*P<*0.001, Fig. S13) exhibit a similar temporal pattern after heat stress: an initial increase in adult population size peaking around day 92, followed by a decline toward day 98.

Sex ratio: Before heat stress, in large populations sex ratio consistent increase over time series (*P*<0.001), whereas small populations showed a steady decline (*P*=0.31, Fig. S14).

**Results for population stability**

*Presence of aggressive male morph reduces population stability*

We report here results based on CV in population size as a measure of population stability (analyses of mean-corrected variability reported in main text). For population stability in the before morph manipulation (BMM) and before heat stress (BHS) phases measured by CV_adult-population-size_ the model comparison among GLMMs showed that neither morph treatment nor time period or their interactions affected stability (Fig.S 6, Table S11). In contrast, stability based on CV_total-population-size_ declined from BMM to BHS phase, and this effect was particularly evident in large, fighter populations (3-way interaction: χ²₁=3.99, *P*=0.05). Across the before (BHS) and after heat stress (AHS) phases, CV_adult-population-size_ was significantly influenced by 3-way interaction, with the greater stability of small, scrambler populations (with respect to small, fighter populations) in the BHS phase disappearing during AHS phase (χ²₁=0.08, *P*=0.01, Fig. S7 A & B, Table S13). CV_total-population-size_ was influenced by the morph treatment and population type interaction with large fighter populations being more unstable than large scrambler populations (χ²₁=7.42, *P*=0.01). Time period significantly interacted with population type, with small populations becoming more unstable in the AHS phase than BHS phase (χ²₁=3.85, *P*=0.05; Fig. S7 C & D, Table S13).

**Figures**

**Figure S1:** Number of emerging fighter males in large and small populations of fighter and scrambler treatment prior to male morph manipulation. The error bars around the mean are standard errors.

**
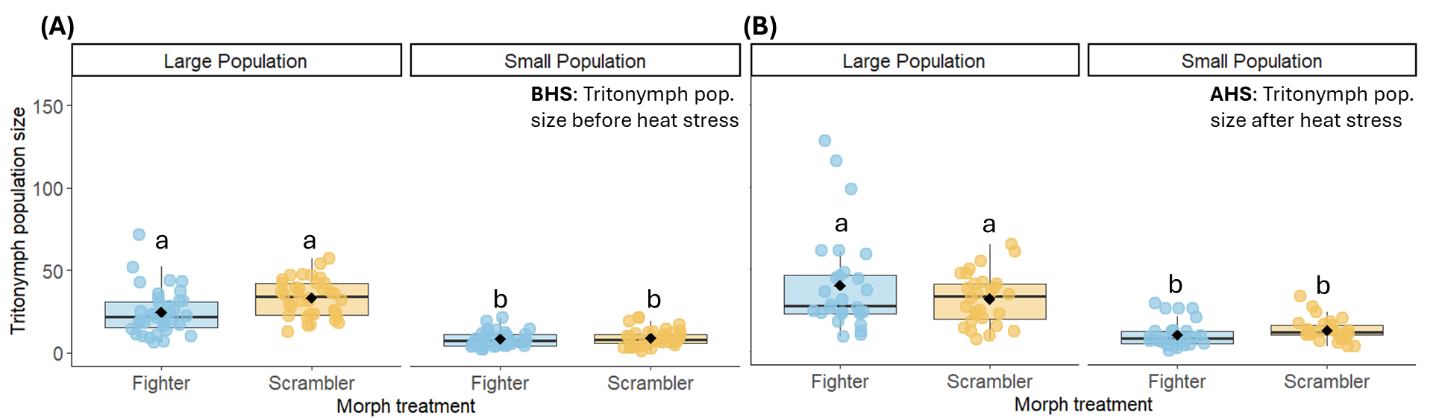
**

**Figure S2:** Number of tritonymphs in the time period (A) BHS (before heat stress) and (B) AHS (after heat stress) in large and small populations of fighter and scrambler treatment. The groups sharing the same letter are not significantly different. The box shows dispersion of data between interquartile range (IQR) of first and third quartiles of data while the line in box represents the median, and whiskers show 1.5 × IQR. The black dot is the mean of the population in that treatment.


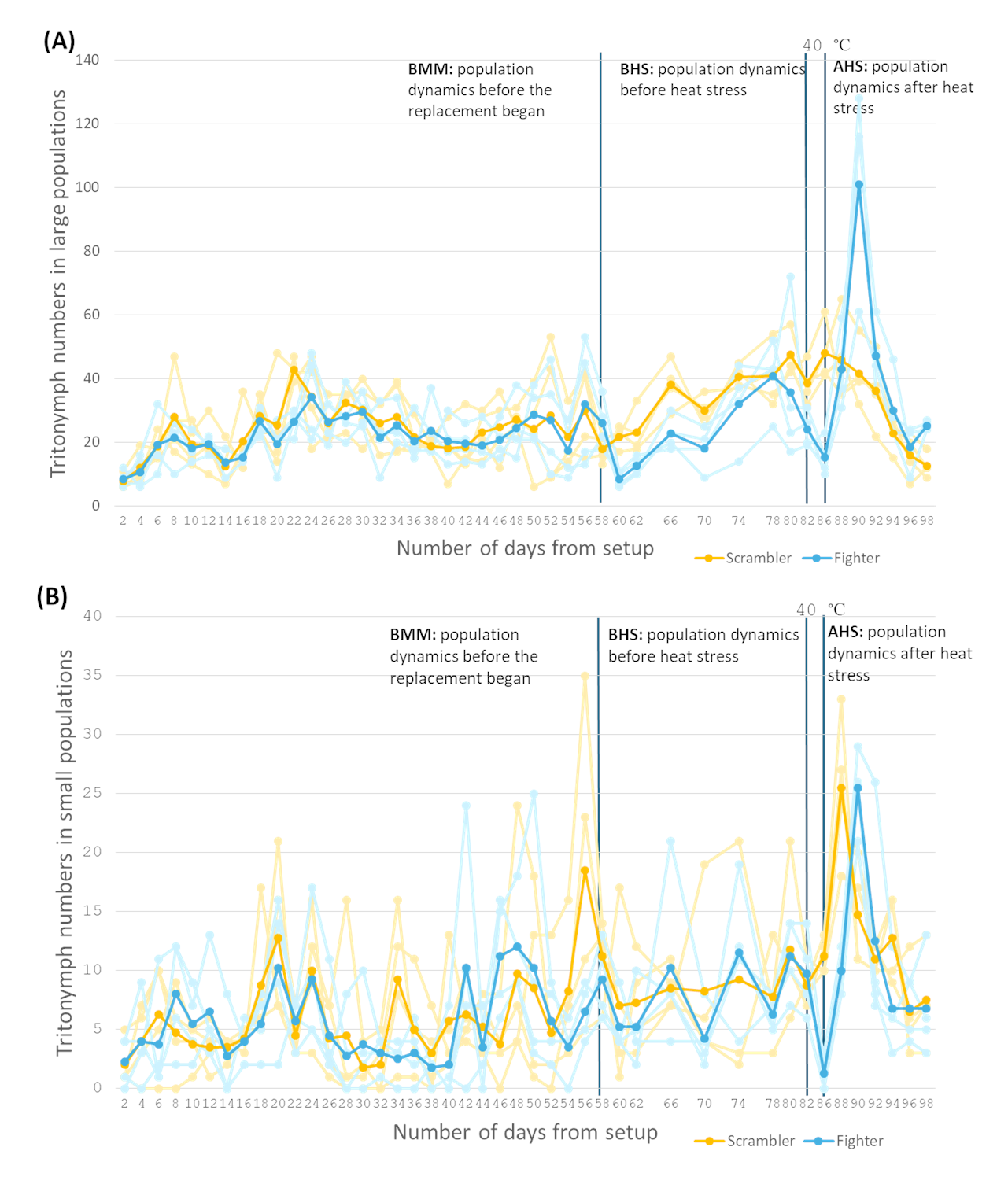


**Figure S3:** Tritonymph population size time series in **(A)** large and **(B)** small populations of the scrambler and fighter treatments. The dark blue color shows the mean tritonymph population size in the fighter male-morph treatment, and the dark orange color represents the mean tritonymph population size in the scrambler male-morph population, while all the lighter colors show tritonymph population size in the respective replicate populations. The fighter and scrambler male-morph treatments were established by replacing male morphs from the stock populations from day 58 from the experiment setup. On day 82 from setup, the populations were given a heat stress for 90 hours at 40°C. Note: Tritonymph census were done on every alternate day except for the period between 62 day and 78 day when census was done every four days.

**
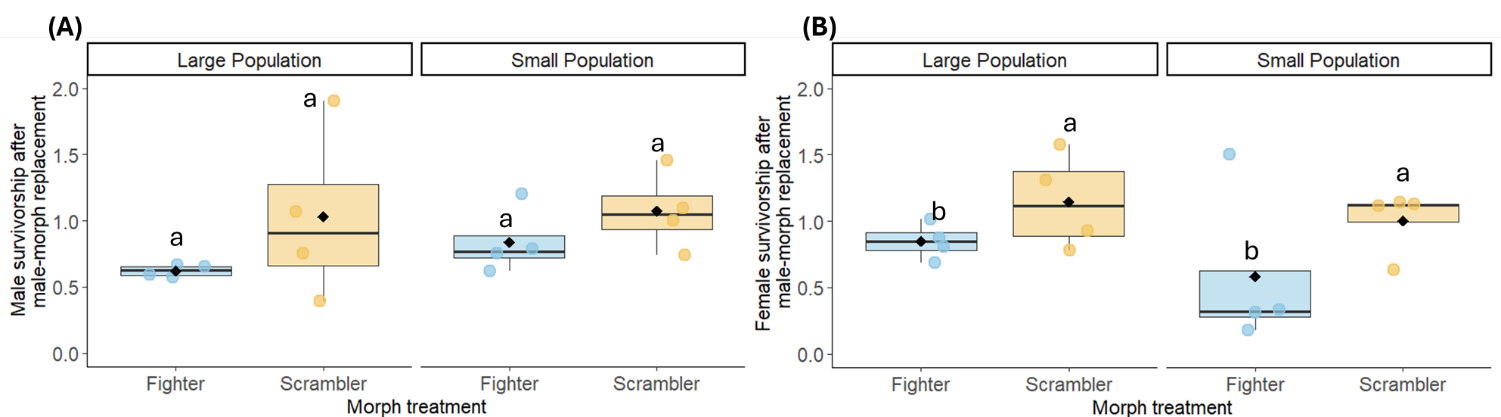
**

**Figure S4:** (A) Decline in male survivorship (day 60) after male morphs were replaced. (B) Decline in female survivorship (day 60) after male morphs were replaced. The groups sharing the same letter are not significantly different. The box shows dispersion of data between interquartile range (IQR) of first and third quartiles of data while the line in box represents the median, and whiskers show 1.5 × IQR. The black dot is the mean of the population in that treatment.

**
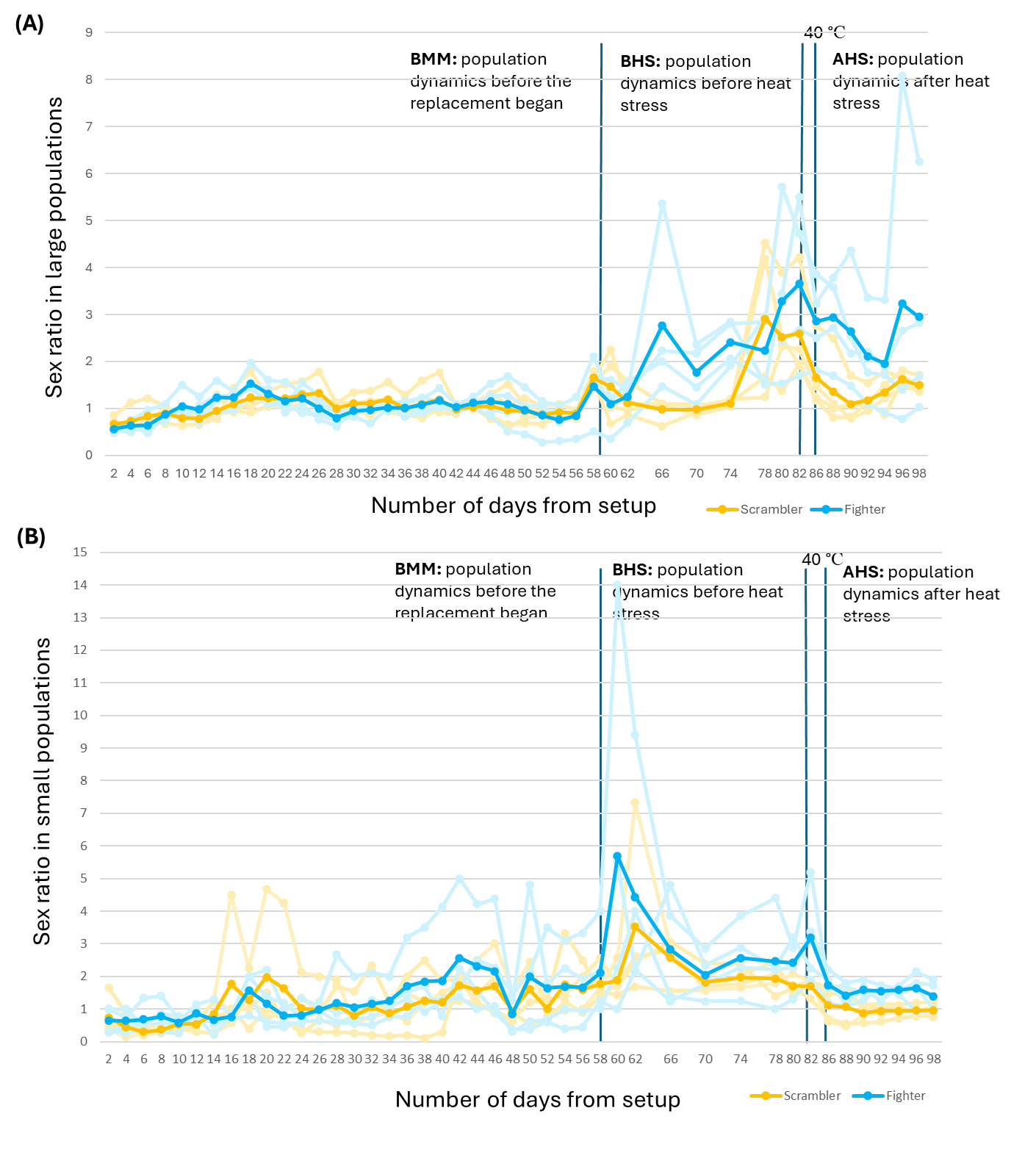
**

**Figure S5:** Sex ratio time series in **(A)** large and **(B)** small populations of scrambler and fighter treatments. The dark blue color shows the mean sex ratio in the fighter male-morph treatment, and the dark orange color represents the mean sex ratio in the scrambler male-morph population, while all the lighter colors show sex ratio in the respective replicate populations. The fighter and scrambler male-morph treatments were established by replacing male morphs from the stock populations from day 58 from the experiment setup. On day 82 from setup, the populations were given a heat wave for 90 hours at 40°C. Note: Population census were done on every alternate day except for the period between 62 day and 78 day when census was done every four days.

**
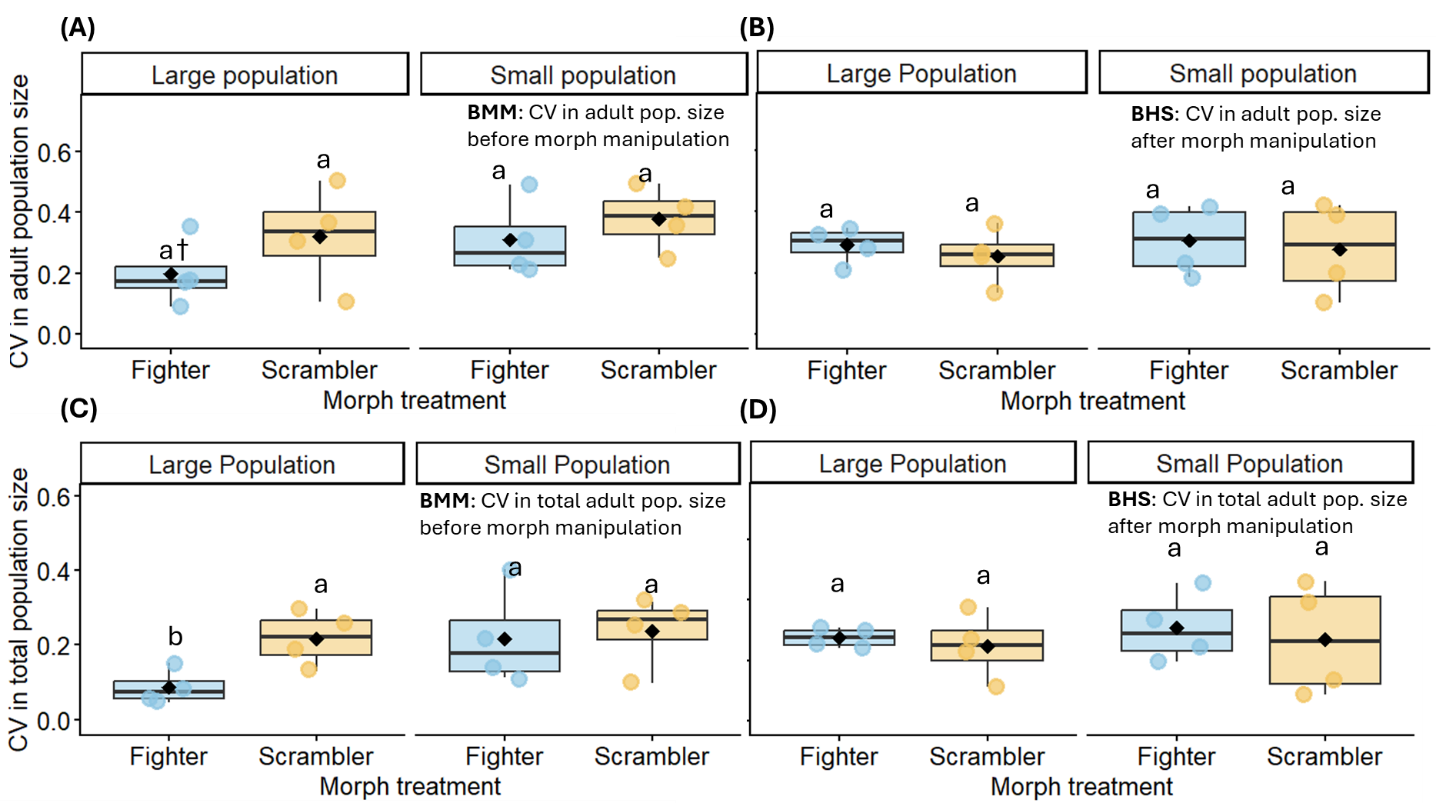
**

**Figure S6:** CV_adult-population-size_ in large and small populations of fighter male-morph and scrambler male-morph treatment (A) in the BMM (day 38 to day 58) (B) and BHS time period. CV_total-population-size_ (adults + tritonymphs) in large and small populations of fighter and scrambler populations treatment (C) in the BMM (day 38 to day 58) (D) and BHS time period. The groups sharing the same letter are not significantly different, but † indicate a marginal trend. The box shows dispersion of data between interquartile range (IQR) of first and third quartiles of data while the line in box represents the median, and whiskers show 1.5 × IQR. The black dot is the mean of the population in that treatment.

**
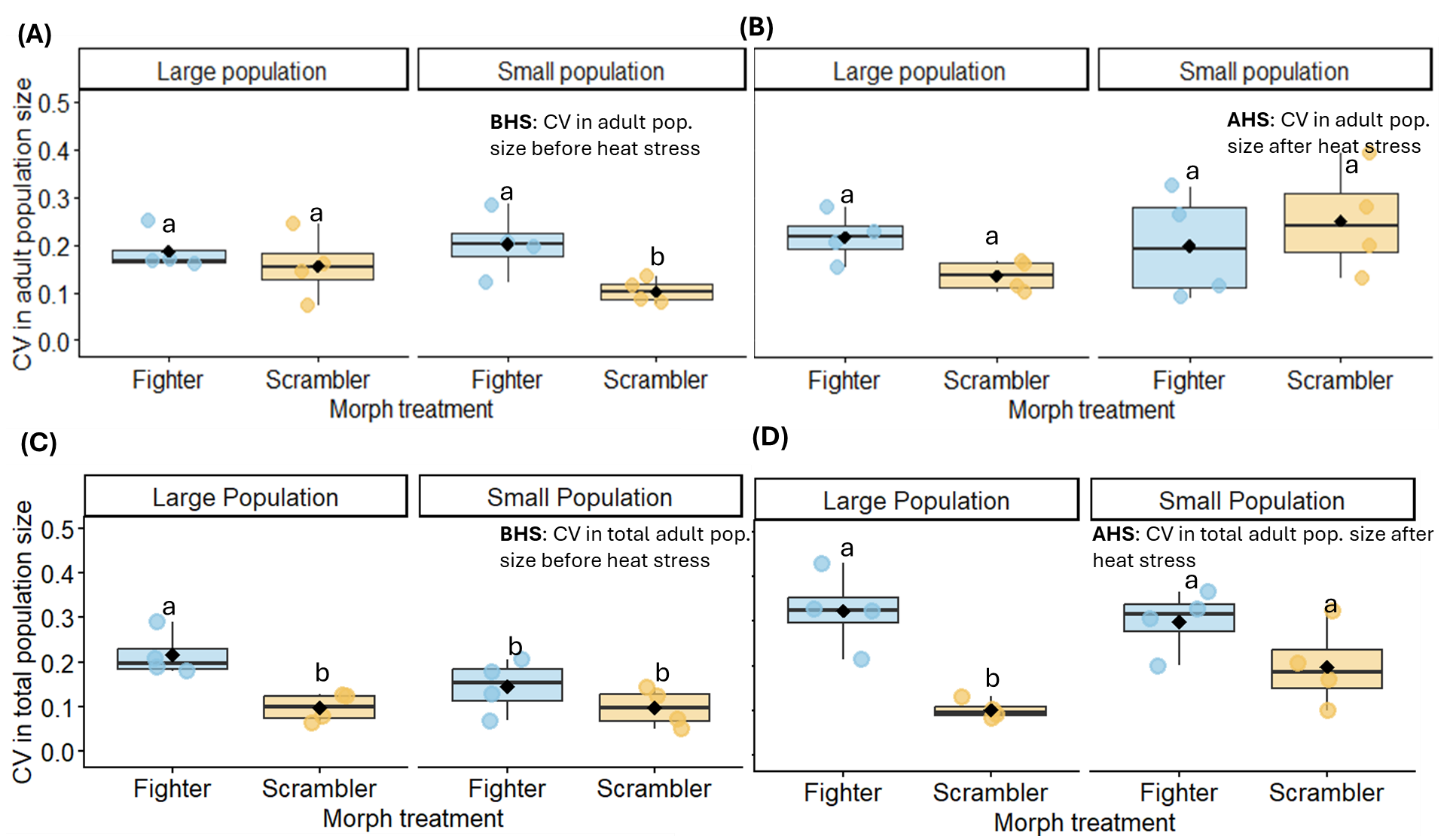
**

**Figure S7:** CV_adult-population-size_ in large and small populations of fighter male-morph and scrambler male-morph treatment (A) in the BHS (B) and AHS time period. CV_total-population-size_ (adults + tritonymphs) in large and small populations of fighter and scrambler populations treatment (C) in the BHS (D) and AHS time period. The groups sharing the same letter are not significantly different. The box shows dispersion of data between interquartile range (IQR) of first and third quartiles of data while the line in box represents the median, and whiskers show 1.5 × IQR. The black dot is the mean of the population in that treatment.


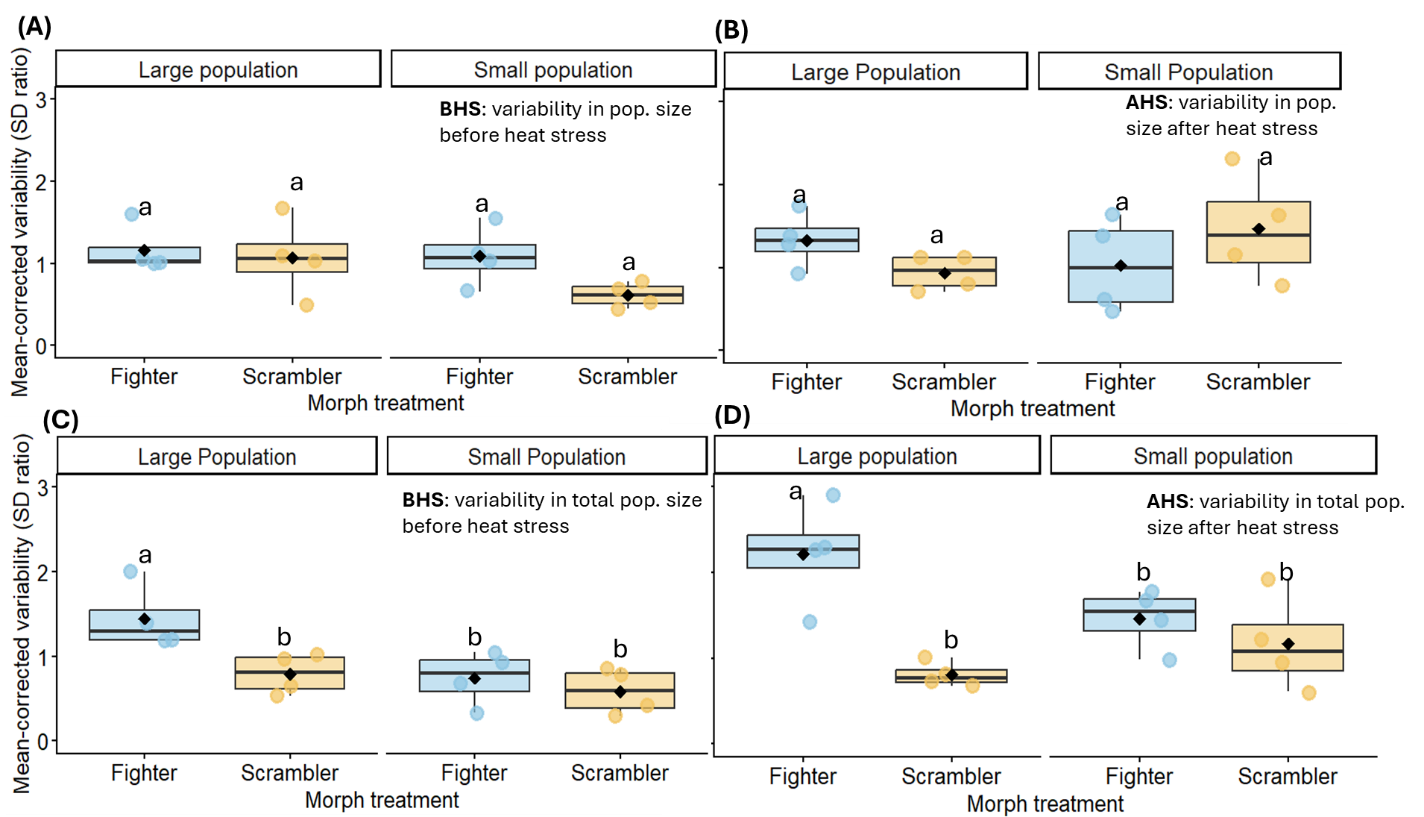


**Figure S8:** Mean-corrected variability_population size_ in large and small populations of fighter and scrambler treatment (A) in the BHS i.e. before the heat stress (B) and AHS time period, and the Mean-corrected variability_population size_ was calculated using adult population size. Mean-corrected variability_total population size_ (adults + tritonymphs) in large and small populations of fighter and scrambler populations treatment (C) in the BHS i.e. before the heat stress (D) and AHS time period, and the Mean-corrected variability_population size_ was calculated using total adult population size. The groups sharing the same letter are not significantly different. The box shows dispersion of data between interquartile range (IQR) of first and third quartiles of data while the line in box represents the median, and whiskers show 1.5 × IQR. The black dot is the mean of the population in that treatment.


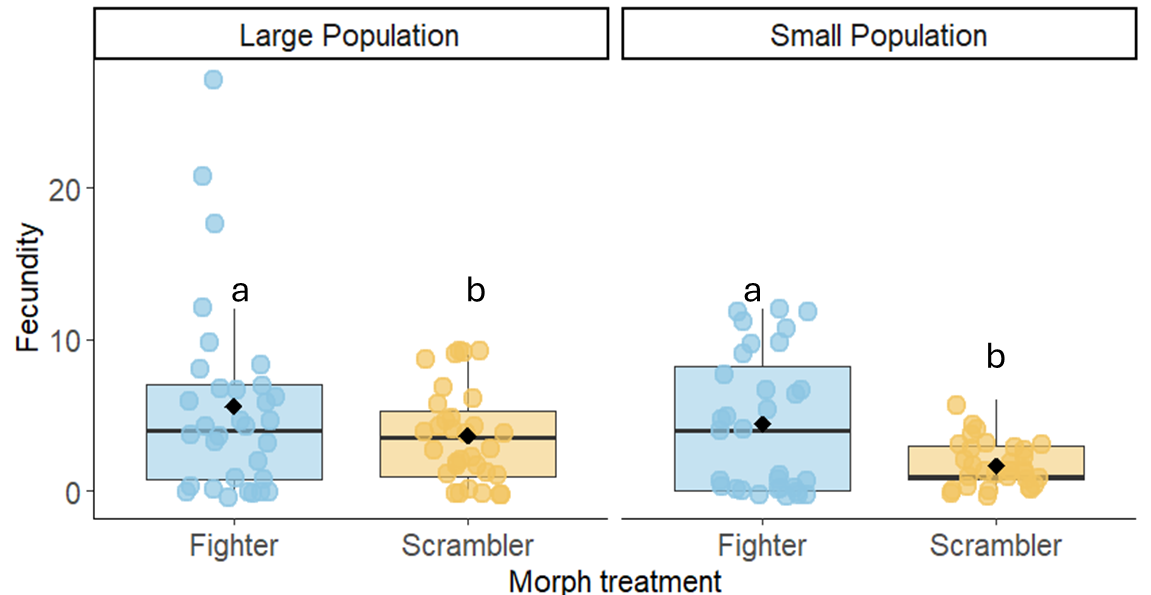


**Figure S9:** Fecundity per female in large and small populations of fighter male-morph and scrambler male-morph treatment after the population dynamics experiment. The females were allowed to lay eggs for 16 hours (n=8 per treatment combination). The groups sharing the same letter are not significantly different. The box shows dispersion of data between interquartile range (IQR) of first and third quartiles of data while the line in box represents the median, and whiskers show 1.5 × IQR. The black dot is the mean of the population in that treatment.


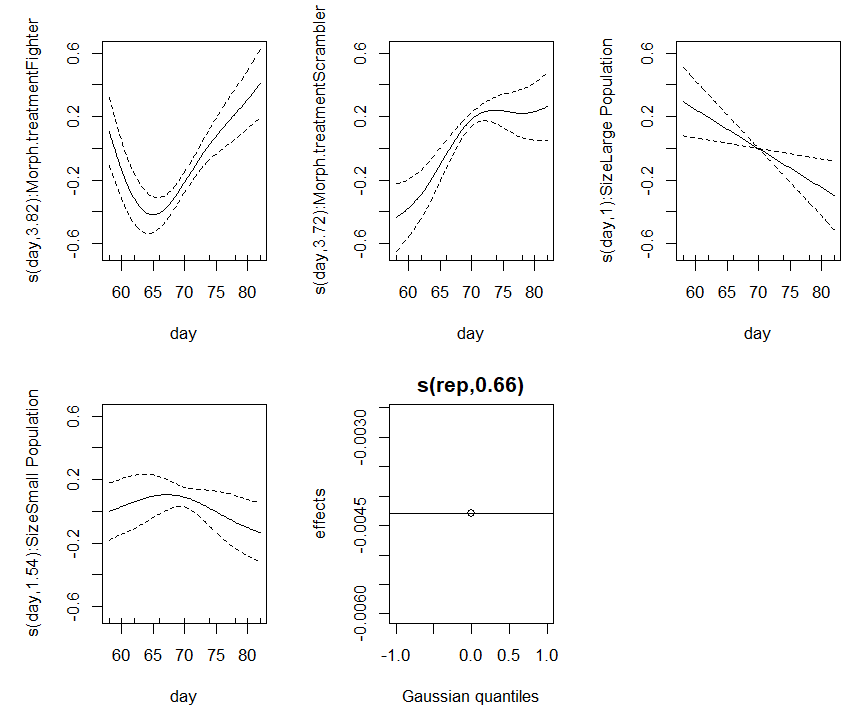


**Figure S10:** Temporal dynamics in adult population size across male morph treatments (fighter, scrambler) and population sizes (large, small) in the before-heat-stress (BHS) period.

**
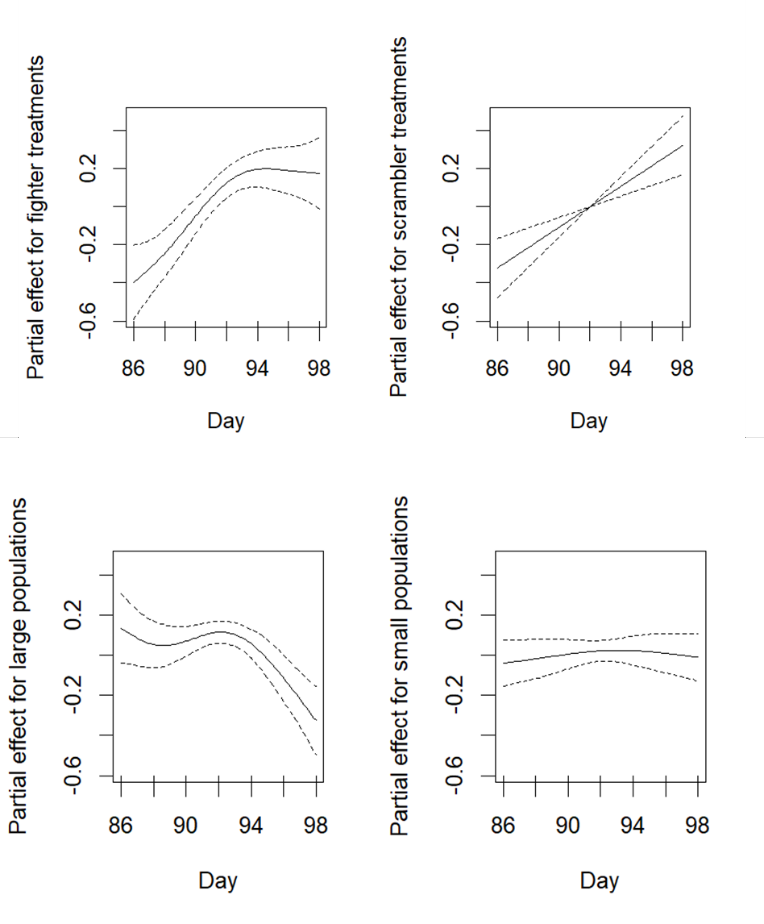
**

**Figure S11:** Temporal dynamics in adult population size across male morph treatments (fighter, scrambler) and population sizes (large, small) in the after-heat-stress (AHS) period.

**
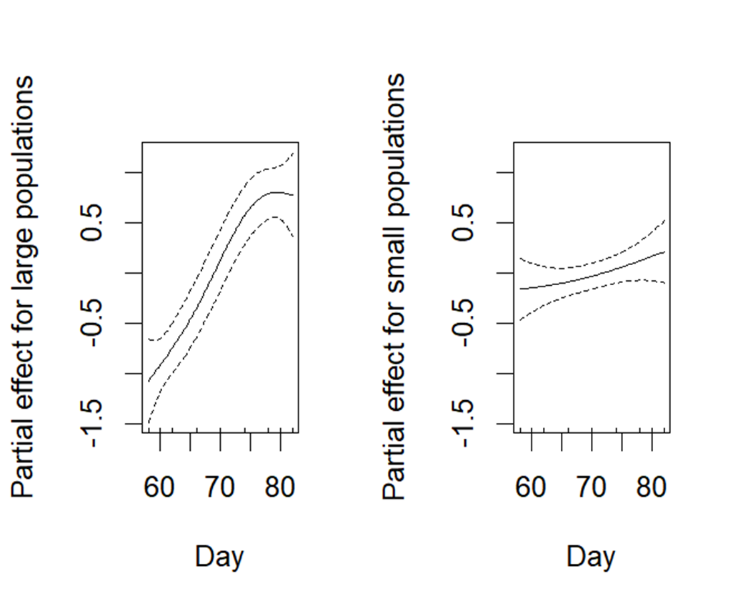
**

**Figure S12:** Temporal dynamics in the tritonymph population across population sizes (large, small) in the before-heat-stress (BHS) period.

**
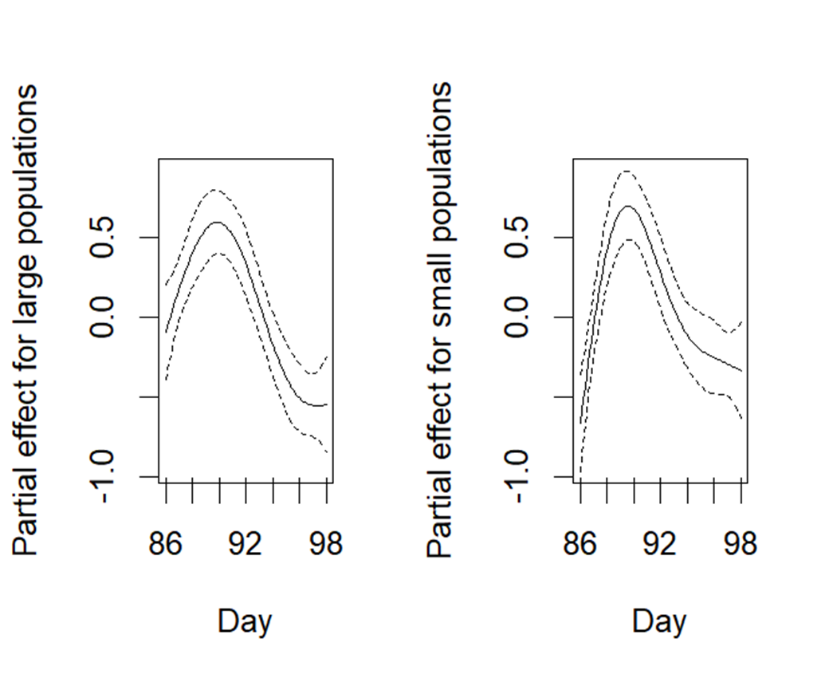
**

**Figure S13:** Temporal dynamics in the tritonymph population across population sizes (large, small) in the after-heat-stress (AHS) period.

**
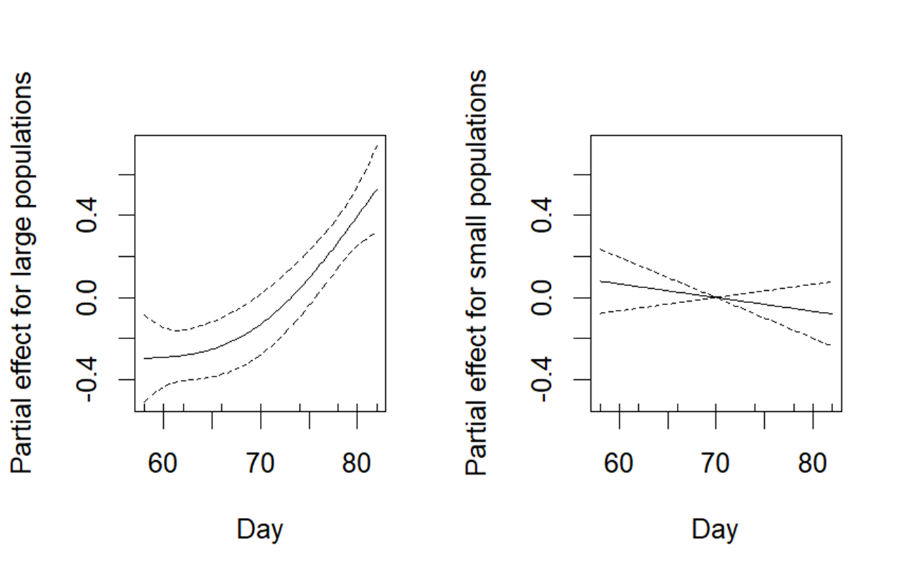
**

**Figure S14:** Temporal dynamics in the sex ration variation across population sizes (large, small) in the before-heat-stress (BHS) period.

**TABLES**

Table S1: Model comparison results for adult population size in the BHS and AHS time periods using nested generalized additive mixed models (GAMMs).

| **Model Comparison** | **Adult population size: BHS time period** | | **Adult population size: AHS time period** | | |  |
| --- | --- | --- | --- | --- | --- | --- |
|  | **Δ Deviance** | ***P*-value** | **Δ Deviance** | ***F*** | ***P*-value** |  |
| Population type | 60.79 | **<0.001** | 18.59 | 4.24 | **0.016** |  |
| Morph treatment | 256.12 | **<0.001** | 34.38 | 28.53 | **<0.001** |  |
| Population type × Morph treatment | 11.51 | **0.001** | 2.14 | 1.028 | 0.31 |  |

Table S2: Coefficients from the best-fitting generalized additive mixed-effects model (GAMM) for adult population size in the BHS and AHS time periods. The tables below present the estimated parametric coefficients and smooth terms from the selected models for BHS and AHS time period.

| **Fixed effects** |  | **Adult population size: BHS time period** | | | | | |  |  | **Adult population size: AHS time period** | | | | |
| --- | --- | --- | --- | --- | --- | --- | --- | --- | --- | --- | --- | --- | --- | --- |
|  | **Estimate** | | | **SE** | ***Z* -value** | ***P*-value** | |  | **Estimate** | | **SE** | | ***t* -value** | ***P*-value** |
| Intercept | | | 4.63 | 0.020 | 224.29 | **<0.001** |  | 4.50 | | | | 0.03 | 137.94 | **<0.001** |
| Population type: small | -1.11 | | | 0.041 | -26.60 | **<0.001** |  | -1.21 | | | | 0.05 | -23.85 | **<0.001** |
| Morph treatment: scrambler | 0.70 | | | 0.020 | 33.65 | **<0.001** |  | 0.87 | | | | 0.03 | 28.14 | **<0.001** |
| Population type: small × Morph treatment: scrambler | -0.14 | | | 0.04 | -3.30 | **<0.001** |  | - | | | | - | - | - |

| **Smooth terms** |  | **Adult population size: BHS time period** | | | | | |  |  | **Adult population size: AHS time period** | | | | |
| --- | --- | --- | --- | --- | --- | --- | --- | --- | --- | --- | --- | --- | --- | --- |
|  | **edf** | | | **Ref.df** | **χ** | ***P*-value** | |  | **edf** | | **Ref.df** | | ***F*** | ***P*-value** |
| Population size: Large | | | 1.00 | 1.00 | 7.51 | **0.006** |  | 3.39 | | | | 3.78 | 8.67 | **<0.001** |
| Population size: Small | 1.54 | | | 2.04 | 9.38 | **0.01** |  | 0.54 | | | | 0.89 | 0.04 | 0.84 |
| Morph treatment: Fighter | 3.82 | | | 3.98 | 178.02 | **<0.001** |  | 2.73 | | | | 3.23 | 7.04 | **<0.001** |
| Morph treatment: Scrambler | 3.72 | | | 3.95 | 92.22 | **<0.001** |  | 1.00 | | | | 1.00 | 17.38 | **<0.001** |
| Replicate | 0.67 | | | 1 | 1.64 | **0.08** |  | 0.59 | | | | 1.00 | 1.34 | 0.12 |

Table S3: Model comparison *F* test results from model comparisons of generalized additive mixed models (GAMMs) for tritonymph population size in the BHS and AHS time periods.

| **Model comparison** | **Tritonymph population size: BHS time period** | | | **Tritonymph population size: AHS time period** | | |  |
| --- | --- | --- | --- | --- | --- | --- | --- |
|  | **Δ Deviance** | ***F*** | ***P*-value** | **Δ Deviance** | ***F*** | ***P*-value** |  |
| Morph treatment × Population type (vs. Morph treatment-only model) | 18.36 | 11.83 | **<0.001** | 3.97 | 9.33 | **<0.001** |  |
| Morph treatment × Population type (vs. Population type-only model) | 5.67 | 7.36 | **0.008** | 2.21 | 9.49 | **0.003** |  |
| Morph treatment × Population type (vs. Morph treatment+ Population type-smooths) | 5.67 | 7.30 | **0.008** | 2.29 | 14.54 | **<0.001** |  |
| Smooths for Morph treatment and Population type | 0.01 | 0.01 | 0.93 | 10.68 | 91.08 | **<0.001** |  |

Table S4: Coefficients from the best-fitting generalized additive mixed-effects model (GAMM) for tritonymph population size in the BHS and AHS time periods. The tables below present the estimated parametric coefficients and smooth terms from the selected models for BHS and AHS time period.

| **Fixed effects** |  | **Tritonymph population size: BHS time period** | | | | |  | | **Tritonymph population size: AHS time period** | | | | |
| --- | --- | --- | --- | --- | --- | --- | --- | --- | --- | --- | --- | --- | --- |
|  | **Estimate** | | | **SE** | ***t* -value** | ***P*-value** | **Estimate** | | | **SE** | | ***t* -value** | ***P*-value** |
| Intercept | | | 4.52 | 0.20 | 22.65 | **<0.001** | | 3.38 | | | 0.12 | 27.39 | **<0.001** |
| Population type: small | -2.83 | | | 0.49 | -5.77 | **<0.001** | | -1.80 | | | 0.30 | -6.05 | **<0.001** |
| Morph treatment: scrambler | 0.50 | | | 0.30 | 1.66 | **0.098** | | -0.34 | | | 0.19 | -1.82 | 0.07 |
| Population type: small × Morph treatment: scrambler | -0.79 | | | 0.29 | -2.78 | **0.006** | | 0.56 | | | 0.18 | 3.06 | **0.002** |

| **Smooth terms** |  | **Tritonymph population size: BHS time period** | | | | | |  |  | **Tritonymph population size: AHS time period** | | | | |
| --- | --- | --- | --- | --- | --- | --- | --- | --- | --- | --- | --- | --- | --- | --- |
|  | **edf** | | | **Ref.df** | ***F*** | ***P*-value** | |  | **edf** | | **Ref.df** | | ***F*** | ***P*-value** |
| Population size: Large | | | 2.38 | 2.87 | 18.73 | **<0.001** |  | 3.34 | | | | 3.76 | 12.11 | **<0.001** |
| Population size: Small | 1.20 | | | 1.38 | 1.63 | 0.26 |  | 3.63 | | | | 3.92 | 11.85 | **<0.001** |
| Replicate | 0.76 | | | 1 | 3.20 | **0.04** |  | 0.67 | | | | 1 | 2.07 | 0.08 |

Table S5: Results from stepwise removal of fixed effects in linear mixed effects model for male and female survivorship once the wrong morphs were replaced with right morphs.

| **Fixed effects** | **Male survivorship after wrong morphs were replaced** | | | **Female survivorship after wrong morphs were replaced** | | |  |
| --- | --- | --- | --- | --- | --- | --- | --- |
|  | **χ** | **d.f.** | ***P*-value** | **χ** | **d.f.** | ***P*-value** |  |
| Population type | 0.61 | 1 | 0.44 | 1.45 | 1 | 0.22 |  |
| Morph treatment | 3.41 | 1 | 0.06 | 4.09 | 1 | **0.04** |  |
| Population type × Morph treatment | 0.28 | 1 | 0.59 | 0.14 | 1 | 0.70 |  |

Table S6: Coefficients from the linear mixed-effects model for male survivorship after morph replacement, and female survivorship after replacement.

| **Fixed effects** |  | **Male survivorship after morph replacement** | | | | |  | | **Female survivorship after morph replacement** | | | | |
| --- | --- | --- | --- | --- | --- | --- | --- | --- | --- | --- | --- | --- | --- |
|  | **Estimate** | | | **SE** | ***Z* -value** | ***P*-value** | **Estimate** | | | **SE** | | ***Z* -value** | ***P*-value** |
| Intercept | | | 0.62 | 0.16 | 3.80 | **0.001** | | 0.85 | | | 0.17 | 5.094 | **<0.001** |
| Population type: small | 0.22 | | | 0.23 | 0.94 | 0.35 | | -0.27 | | | 0.24 | -1.14 | 0.25 |
| Morph treatment: scrambler | 0.41 | | | 0.23 | 1.77 | 0.08 | | 0.30 | | | 0.24 | 1.27 | 0.21 |
| Population type: small × Morph treatment: scrambler | -0.17 | | | 0.37 | -0.54 | 0.59 | | 0.13 | | | 0.33 | 0.38 | 0.71 |

Table S7: Results of *F* tests comparing nested generalized additive mixed models (GAMMs) for log-transformed sex ratio in the BHS and AHS time periods.

| **Model Comparison** | **Sex ratio: BHS time period** | | | **Sex ratio: AHS time period** | | |  |
| --- | --- | --- | --- | --- | --- | --- | --- |
|  | **Δ Deviance** | ***F*** | ***P*-value** | **Δ Deviance** | ***F*** | ***P*-value** |  |
| Morph treatment (vs. Population type-only model) | 0.41 | 20.52 | **0.01** | - | - | **-** |  |
| Population type smoother (vs. morph-only model) | 5.15 | 10.00 | **<0.001** | 0.44 | 1.36 | 0.26 |  |
| Morph treatment × Population type (vs. Morph treatment + Population type smooths) | 0.00 | 0.00 | 0.97 | 0.02 | -0.17 | -0.68 |  |
| Morph treatment smoother (vs. Population type smoother only) | 0.22 | 0.95 | 0.33 | 0.21 | 1.45 | 0.23 |  |
| Temporal smoother (vs. linear model) | - | - | - | 0.05 | 0.31 | 0.58 |  |

Table S8: Coefficients from the best-fitting generalized additive mixed-effects model (GAMM) for sex ratio in the BHS and AHS time periods. The tables below present the estimated parametric coefficients and smooth terms from the selected models for BHS and AHS time period.

| **Fixed effects** |  | **Sex ratio: BHS time period** | | | | |  | | **Sex ratio: AHS time period** | | | | |
| --- | --- | --- | --- | --- | --- | --- | --- | --- | --- | --- | --- | --- | --- |
|  | **Estimate** | | | **SE** | ***t* -value** | ***P*-value** | **Estimate** | | | **SE** | | **Z -value** | ***P*-value** |
| Intercept | | | 0.70 | 0.09 | 7.57 | **<0.001** | | 0.82 | | | 0.13 | 6.53 | **<0.001** |
| Population type: small | 0.48 | | | 0.21 | 2.24 | **0.03** | | -0.39 | | | 0.14 | -3.60 | **<0.001** |
| Morph treatment: scrambler | -0.14 | | | 0.13 | -1.13 | 0.26 | | -0.52 | | | 0.14 | -2.68 | **0.01** |
| Population type: small × Morph treatment: scrambler | - | | | - | - | - | | - | | | - | - | - |

| **Smooth terms** |  | **Sex ratio: BHS time period** | | | | | |  |  | **Sex ratio: AHS time period** | | | | |
| --- | --- | --- | --- | --- | --- | --- | --- | --- | --- | --- | --- | --- | --- | --- |
|  | **edf** | | | **Ref.df** | ***F*** | ***P*-value** | |  | **edf** | | **Ref.df** | | ***F*** | ***P*-value** |
| Population size: Large | | | 2.++ | 2.44 | 12.78 | **<0.001** |  | - | | | | - | - | **-** |
| Population size: Small | 1.00 | | | 1.00 | 1.04 | 0.31 |  | - | | | | - | - | **-** |
| Replicate | 0.49 | | | 1.00 | 0.97 | 0.16 |  | - | | | | - | - | - |

Table S9: Results from stepwise removal of fixed effects in linear mixed effects model for decline in adult population size after heat stress.

| **Fixed effects** | **Decline in adult population size** | | |
| --- | --- | --- | --- |
|  | **χ** | **d.f.** | ***P*-value** |
| Population type | - | - | - |
| Morph treatment | - | - | - |
| Population type × Morph treatment | 4.36 | 1 | **0.04** |

Table S10: Coefficients from the linear mixed-effects model for decline in population size after heat stress.

| **Fixed effects** |  | **Decline in adult population size after heat stress** | | | | | | |  |
| --- | --- | --- | --- | --- | --- | --- | --- | --- | --- |
|  | **Estimate** | | **SE** | | **Z -value** | | ***P*-value** | | |
| Intercept | 0.60 | | 0.09 | 6.86 | | | | **<0.001** | |
| Population type: small | -0.02 | | 0.12 | -0.16 | | | | 0.87 | |
| Morph treatment: scrambler | 0.40 | | 0.12 | 3.22 | | | | **0.001** | |
| Population type: small × Morph treatment: scrambler | -0.39 | | 0.17 | | | -2.24 | | **0.03** | |

Table S11: Results from stepwise removal of fixed effects in generalized mixed effects model using gamma error structure for CV_adult-population-size_ and CV_total-population-size_ before and after morph manipulation.

| **Fixed effects** | CV_adult-population-size_ | | | CV_total-population-size_ | | |  |
| --- | --- | --- | --- | --- | --- | --- | --- |
|  | **χ** | **d.f.** | ***P*-value** | **χ** | **d.f.** | ***P*-value** |  |
| Population type | 1.24 | 1 | 0.27 | 1.58 | 1 | 0.21 |  |
| Morph treatment | 0.51 | 1 | 0.47 | 0.27 | 1 | 0.61 |  |
| Time period | 0.09 | 1 | 0.77 | 4.22 | 1 | **0.04** |  |
| Population type × Morph treatment | 0.20 | 1 | 0.66 | 1.38 | 1 | 0.24 |  |
| Population type × Time period | 1.37 | 1 | 0.24 | 1.34 | 1 | 0.25 |  |
| Morph treatment× Time period | 3.61 | 1 | 0.06 | 3.43 | 1 | 0.06 |  |
| Population type × Morph treatment× Time period | 0.38 | 1 | 0.54 | 3.99 | 1 | 0.05 |  |

Table S12: Coefficients from the generalized mixed-effects model for CV_adult-population-size_ and CV_total-population-size_ before and after morph manipulation. For CV_adult-population-size_, the variance explained by random effect, i.e., population identity was 0.044. For CV_total-population-size_, the variance explained by population identity was 0.217.

| **Fixed effects** |  | CV_adult-population-size_ | | | | |  | | CV_total-population-size_ | | | | |
| --- | --- | --- | --- | --- | --- | --- | --- | --- | --- | --- | --- | --- | --- |
|  | **Estimate** | | | **SE** | **Z -value** | ***P*-value** | **Estimate** | | | **SE** | | **Z -value** | ***P*-value** |
| Intercept | | | -1.23 | 0.20 | -6.22 | **<0.001** | | 3.70 | | | 0.79 | 4.65 | **<0.001** |
| Population type: small | 0.03 | | | 0.28 | 0.09 | 0.92 | | -0.38 | | | 1.05 | -0.36 | 0.72 |
| Morph treatment: scrambler | -0.13 | | | 0.28 | -0.46 | 0.64 | | 0.41 | | | 1.16 | 0.35 | 0.73 |
| Time Period: BMM | -0.44 | | | 0.24 | -1.82 | 0.07 | | 7.88 | | | 2.49 | 3.17 | **0.001** |
| Population type: small × Morph treatment: scrambler | 0.01 | | | 0.40 | 0.01 | 0.99 | | 0.06 | | | 1.57 | 0.04 | 0.97 |
| Population type: small × Time Period: BMM | 0.45 | | | 0.38 | 1.32 | 0.19 | | -6.55 | | | 2.74 | -2.39 | **0.02** |
| Morph treatment: scrambler × Time Period: BMM | 0.64 | | | 0.34 | 1.89 | 0.06 | | -7.33 | | | 2.78 | -2.63 | **0.008** |
| Population type: small × Morph treatment: scrambler × Time Period: BMM | -0.30 | | | 0.48 | -0.62 | 0.54 | | 6.48 | | | 3.22 | 2.01 | **0.04** |

Table S13: Results from stepwise removal of fixed effects in generalized mixed effects model using gamma error structure for CV_adult-population-size_ and CV_total-population-size_ size before and after heat stress.

| **Fixed effects** | CV_adult-population-size_ | | | CV_total-population-size_ | | |  |
| --- | --- | --- | --- | --- | --- | --- | --- |
|  | **χ** | **d.f.** | ***P*-value** | **χ** | **d.f.** | ***P*-value** |  |
| Population type | 3.45 | 1 | 0.56 | - | - | - |  |
| Morph treatment | 2.32 | 1 | 0.13 | - | - | - |  |
| Time period | 2.41 | 1 | 0.12 | - | - | - |  |
| Population type × Morph treatment | 0.61 | 1 | 0.44 | 7.42 | 1 | **0.01** |  |
| Population type × Time period | 1.55 | 1 | 0.21 | 3.85 | 1 | **0.05** |  |
| Morph treatment× Time period | 2.02 | 1 | 0.16 | 0.08 | 1 | 0.78 |  |
| Population type × Morph treatment× Time period | 0.08 | 1 | **0.006** | 0.81 | 1 | 0.37 |  |

Table S14: Coefficients from the generalized mixed-effects model for CV_adult-population-size_ and CV_total-population-size_. For CV_adult-population-size_, the variance explained by random effect, i.e., population identity was 0.841. For CV_total-population-size_, the variance explained by population identity was <0.001.

| **Fixed effects** |  | CV_adult-population-size_ | | | | |  | | CV_total-population-size_ | | | | |
| --- | --- | --- | --- | --- | --- | --- | --- | --- | --- | --- | --- | --- | --- |
|  | **Estimate** | | | **SE** | **Z -value** | ***P*-value** | **Estimate** | | | **SE** | | **Z -value** | ***P*-value** |
| Intercept | | | 4.73 | 0.80 | 5.93 | **<0.001** | | 3.09 | | | 0.48 | 6.51 | **<0.001** |
| Population type: small | 0.49 | | | 1.16 | 0.42 | 0.67 | | 0.25 | | | 0.70 | 0.36 | 0.72 |
| Morph treatment: scrambler | 2.71 | | | 1.38 | 1.97 | **0.05** | | 6.80 | | | 1.59 | 4.27 | **<0.001** |
| Time Period: BHS | 0.73 | | | 0.99 | 0.73 | 0.46 | | 1.54 | | | 0.85 | 1.80 | 0.07 |
| Population type: small × Morph treatment: scrambler | -3.72 | | | 1.77 | -2.10 | **0.04** | | -5.09 | | | 1.84 | -2.76 | **0.01** |
| Population type: small × Time Period: BHS | -0.80 | | | 1.39 | -0.57 | 0.57 | | 2.01 | | | 1.45 | 1.38 | 0.16 |
| Morph treatment: scrambler × Time Period: BHS | -1.60 | | | 1.68 | -0.95 | 0.34 | | -1.15 | | | 2.35 | -0.49 | 0.62 |
| Population type: small × Morph treatment: scrambler × Time Period: BHS | 7.27 | | | 2.45 | 2.97 | **0.002** | | 2.85 | | | 3.16 | 0.90 | 0.37 |

Table S15: Results from stepwise removal of fixed effects in generalized mixed effects model using gaussian error structure for Mean-corrected variability_adult-population-size_ and Mean-corrected variability_total-population-size_ before and after morph manipulation.

| **Fixed effects** | Mean-corrected variability_adult-population-size_ | | | Mean-corrected variability_total-population-size_ | | |  |
| --- | --- | --- | --- | --- | --- | --- | --- |
|  | **χ** | **d.f.** | ***P*-value** | **χ** | **d.f.** | ***P*-value** |  |
| Population type | 0.04 | 1 | 0.84 | 0.12 | 1 | 0.72 |  |
| Morph treatment | 0.60 | 1 | 0.44 | - | - | - |  |
| Time period | 0.00 | 1 | 0.96 | - | - | **-** |  |
| Population type × Morph treatment | 0.27 | 1 | 0.60 | 2.63 | 1 | 0.10 |  |
| Population type × Time period | 1.55 | 1 | 0.21 | 1.99 | 1 | 0.16 |  |
| Morph treatment× Time period | 2.06 | 1 | 0.15 | 4.60 | 1 | **0.03** |  |
| Population type × Morph treatment× Time period | 0.17 | 1 | 0.68 | 1.50 | 1 | 0.22 |  |

Table S16: Coefficients from the generalized mixed-effects model for Mean-corrected variability_adult-population-size_ and Mean-corrected variability_total-population-size_ before and after morph manipulation. For Mean-corrected variability_adult-population-size_, the variance explained by random effect, i.e., population identity was 0.0012. For Mean-corrected variability_total-population-size_, the variance explained by population identity was 0.006.

| **Fixed effects** |  | Mean-corrected variability_adult-population-size_ | | | | |  | | Mean-corrected variability_total-population-size_ | | | | |
| --- | --- | --- | --- | --- | --- | --- | --- | --- | --- | --- | --- | --- | --- |
|  | **Estimate** | | | **SE** | **Z -value** | ***P*-value** | **Estimate** | | | **SE** | | **Z -value** | ***P*-value** |
| Intercept | | | 0.12 | 0.21 | 0.57 | **0.57** | | 0.37 | | | 0.22 | 1.68 | **0.09** |
| Population type: small | -0.14 | | | 0.30 | 0.47 | 0.64 | | -0.09 | | | 0.30 | -0.30 | 0.77 |
| Morph treatment: scrambler | -0.08 | | | 0.30 | -0.26 | 0.79 | | -0.10 | | | 0.30 | -0.32 | 0.75 |
| Time Period: BMM | -0.47 | | | 0.30 | -1.60 | 0.10 | | -1.24 | | | 0.30 | -4.07 | **<0.001** |
| Population type: small × Morph treatment: scrambler | -0.03 | | | 0.42 | -0.07 | 0.93 | | -0.14 | | | 0.43 | -0.32 | 0.74 |
| Population type: small × Time Period: BMM | 0.49 | | | 0.42 | 1.19 | 0.23 | | 0.83 | | | 0.43 | 1.92 | **0.05** |
| Morph treatment: scrambler × Time Period: BMM | 0.56 | | | 0.42 | 1.33 | 0.18 | | 1.08 | | | 0.43 | 2.50 | **0.01** |
| Population type: small × Morph treatment: scrambler × Time Period: BMM | -0.25 | | | 0.59 | -0.41 | 0.67 | | -0.76 | | | 0.61 | -1.24 | 0.21 |

Table S17: Results from stepwise removal of fixed effects in generalized mixed effects model using gamma error structure for Mean-corrected variability_adult-population-size_ and Mean-corrected variability_total-population-size_ before and after heat stress.

| **Fixed effects** | Mean-corrected variability_adult-population-size_ | | | Mean-corrected variability_total-population-size_ | | |  |
| --- | --- | --- | --- | --- | --- | --- | --- |
|  | **χ** | **d.f.** | ***P*-value** | **χ** | **d.f.** | ***P*-value** |  |
| Population type | 1.01 | 1 | 0.31 | - | - | - |  |
| Morph treatment | 1.32 | 1 | 0.25 | - | - | - |  |
| Time period | 1.74 | 1 | 0.19 | - | - | - |  |
| Population type × Morph treatment | 0.46 | 1 | 0.50 | 5.10 | 1 | **0.02** |  |
| Population type × Time period | 1.35 | 1 | 0.25 | 4.21 | 1 | **0.04** |  |
| Morph treatment× Time period | 2.13 | 1 | 0.14 | 0.79 | 1 | 0.37 |  |
| Population type × Morph treatment× Time period | 5.46 | 1 | **0.02** | 0.53 | 1 | 0.47 |  |

Table S18: Coefficients from the generalized mixed-effects model for Mean-corrected variability_adult-population-size_ and Mean-corrected variability_total-population-size_ before and after heat stress. For Mean-corrected variability_adult-population-size_, the variance explained by random effect, i.e., population identity was <0.001. For Mean-corrected variability_total-population-size_, the variance explained by population identity was <0.001.

| **Fixed effects** |  | Mean-corrected variability_adult-population-size_ | | | | |  | | Mean-corrected variability_total-population-size_ | | | | |
| --- | --- | --- | --- | --- | --- | --- | --- | --- | --- | --- | --- | --- | --- |
|  | **Estimate** | | | **SE** | **Z -value** | ***P*-value** | **Estimate** | | | **SE** | | **Z -value** | ***P*-value** |
| Intercept | | | 0.26 | 0.17 | 1.54 | 0.12 | | 0.76 | | | 0.16 | 4.72 | **<0.001** |
| Population type: small | -0.37 | | | 0.24 | -1.54 | 0.12 | | -0.41 | | | 0.23 | -1.79 | 0.07 |
| Morph treatment: scrambler | -0.35 | | | 0.24 | -1.45 | 0.14 | | -0.99 | | | 0.23 | -4.34 | **<0.001** |
| Time Period: BHS | -0.13 | | | 0.24 | -0.55 | 0.59 | | -0.42 | | | 0.23 | -1.83 | 0.06 |
| Population type: small × Morph treatment: scrambler | 0.76 | | | 0.34 | 2.25 | **0.02** | | 0.71 | | | 0.32 | 2.19 | **0.02** |
| Population type: small × Time Period: BHS | 0.28 | | | 0.34 | 0.82 | 0.41 | | -0.32 | | | 0.32 | -0.10 | 0.32 |
| Morph treatment: scrambler × Time Period: BHS | 0.12 | | | 0.34 | 0.58 | 0.56 | | 0.37 | | | 0.32 | 1.15 | 0.25 |
| Population type: small × Morph treatment: scrambler × Time Period: BHS | -1.16 | | | 0.48 | -2.44 | **0.01** | | -0.33 | | | 0.46 | -0.73 | 0.47 |

Table S19: Results from stepwise removal of fixed effects in linear mixed-effects for maximum and realized rate of growth before and after heat stress.

| **Fixed effects** | **Maximum rate of growth** | | | **Realized rate of growth** | | |  |
| --- | --- | --- | --- | --- | --- | --- | --- |
|  | **χ** | **d.f.** | ***P*-value** | **χ** | **d.f.** | ***P*-value** |  |
| Population type | - | - | - | - | - | - |  |
| Morph treatment | - | - | - | - | - | - |  |
| Time period | 4.00 | 1 | **0.05** | 14.07 | 1 | **<0.001** |  |
| Population type × Morph treatment | 4.01 | 1 | **0.05** | 5.25 | 1 | **0.02** |  |
| Population type × Time period | 1.52 | 1 | 0.22 | 0.46 | 1 | 0.50 |  |
| Morph treatment× Time period | 0.44 | 1 | 0.51 | 1.89 | 1 | 0.17 |  |
| Population type × Morph treatment× Time period | 0.11 | 1 | 0.74 | 0.22 | 1 | 0.64 |  |

Table S20: Coefficients from the linear mixed-effects model for maximum and realized rate of growth. For maximum rate of growth, the variance explained by random effect, i.e., population identity was <0.001. For realized rate of growth, the variance explained by population identity was <0.001.

| **Fixed effects** |  | **Maximum rate of growth** | | | | |  | **Realized rate of growth** | | | | |
| --- | --- | --- | --- | --- | --- | --- | --- | --- | --- | --- | --- | --- |
|  | **Estimate** | | | **SE** | **Z -value** | ***P*-value** | **Estimate** | | **SE** | | **Z -value** | ***P*-value** |
| Intercept | | | 1.51 | 0.10 | 15.29 | **<0.001** | 1.76 | | | 0.15 | 11.35 | **<0.001** |
| Population type: small | 0.06 | | | 0.14 | 0.43 | 0.67 | -0.14 | | | 0.22 | -0.63 | 0.53 |
| Morph treatment: scrambler | 0.27 | | | 0.14 | -1.95 | 0.05 | -0.33 | | | 0.22 | -1.52 | 0.13 |
| Time Period: BHS | <0.001 | | | 0.14 | 0 | 1 | -0.34 | | | 0.22 | -1.55 | 0.12 |
| Population type: small × Morph treatment: scrambler | 0.24 | | | 0.20 | 1.24 | 0.22 | 0.63 | | | 0.31 | 2.04 | 0.04 |
| Population type: small × Time Period: BHS | 0.22 | | | 0.20 | -1.13 | 0.26 | -0.05 | | | 0.31 | -0.15 | 0.88 |
| Morph treatment: scrambler × Time Period: BHS | 0.14 | | | 0.20 | -0.71 | 0.48 | -0.21 | | | 0.31 | -0.67 | 0.50 |
| Population type: small × Morph treatment: scrambler × Time Period: BHS | 0.09 | | | 0.28 | 0.34 | 0.74 | -0.20 | | | 0.44 | -0.47 | 0.64 |

Table S21: Results from stepwise removal of fixed effects in linear mixed-effects model for percentage recovery of populations after heat stress.

| **Fixed effects** | **Percentage recovery after heat stress** | | |
| --- | --- | --- | --- |
|  | **χ** | **d.f.** | ***P*-value** |
| Population type | 0.04 | 1 | 0.85 |
| Morph treatment | 8.03 | 1 | **0.004** |
| Population type × Morph treatment | 0.25 | 1 | 0.62 |

Table S22: Coefficients from the linear mixed-effects model for percentage recovery after heat stress.

| **Fixed effects** |  | **Percentage recovery after heat stress** | | | | | | |  |
| --- | --- | --- | --- | --- | --- | --- | --- | --- | --- |
|  | **Estimate** | | **SE** | | **Z -value** | | ***P*-value** | | |
| Intercept | 70.93 | | 12.35 | 5.75 | | | | **<0.001** | |
| Population type: small | 8.59 | | 17.46 | 0.49 | | | | 0.62 | |
| Morph treatment: scrambler | 46.38 | | 17.46 | 2.66 | | | | **0.01** | |
| Population type: small × Morph treatment: scrambler | -12.42 | | 24.69 | | | -0.50 | | 0.62 | |

Table S23: Results from stepwise removal of fixed effects in generalized mixed-effects model with negative binomial error structure for fecundity.

| **Fixed effects** | **Fecundity after heat stress** | | |
| --- | --- | --- | --- |
|  | **χ** | **d.f.** | ***P*-value** |
| Population type | 5.42 | 1 | **0.02** |
| Morph treatment | 10.49 | 1 | **0.001** |
| Population type × Morph treatment | 1.78 | 1 | 0.18 |

Table S24: Coefficients from the generalized mixed-effects model for fecundity. For fecundity, the variance explained by random effect, i.e., population identity was <0.001.

| **Fixed effects** |  | **Fecundity after population dynamics experiment** | | | | | | |  |
| --- | --- | --- | --- | --- | --- | --- | --- | --- | --- |
|  | **Estimate** | | **SE** | | **Z -value** | | ***P*-value** | | |
| Intercept | 1.72 | | 0.19 | 9.01 | | | | **<0.001** | |
| Population type: small | -0.23 | | 0.27 | -0.85 | | | | 0.40 | |
| Morph treatment: scrambler | -0.43 | | 0.28 | -1.54 | | | | 0.12 | |
| Population type: small × Morph treatment: scrambler | -0.54 | | 0.40 | | | -1.34 | | 0.18 | |
